# Longitudinal association between DNA methylation and type 2 diabetes: findings from the KORA F4/FF4 study

**DOI:** 10.1186/s12933-024-02558-8

**Published:** 2025-01-18

**Authors:** Liye Lai, Dave Laurence Juntilla, Monica Del C Gomez-Alonso, Harald Grallert, Barbara Thorand, Aiman Farzeen, Wolfgang Rathmann, Juliane Winkelmann, Holger Prokisch, Christian Gieger, Christian Herder, Annette Peters, Melanie Waldenberger

**Affiliations:** 1 Research Unit Molecular Epidemiology, Helmholtz Zentrum München, German Research Center for Environmental Health (GmbH), Neuherberg, Germany; 2 Institute of Epidemiology, Helmholtz Zentrum München, German Research Center for Environmental Health (GmbH), Neuherberg, Germany; 3 Institute for Medical Information Processing, Biometry, and Epidemiology (IBE), Pettenkofer School of Public Health, Faculty of Medicine, Ludwig Maximilians University, Munich, Germany; 4 German Center for Diabetes Research (DZD), Neuherberg, Germany; 5 Institute of Neurogenomics, Computational Health Center, Helmholtz Zentrum München, Neuherberg, Germany; 6 Institute for Biometrics and Epidemiology, German Diabetes Center, Leibniz Center for Diabetes Research, Heinrich Heine University Düsseldorf, Düsseldorf, Germany; 7 Institute of Human Genetics, School of Medicine, Technical University Munich, Munich, Germany; 8 Cluster for Systems Neurology (SyNergy), Munich, Germany; 9 Chair of Neurogenetics, Technische Universität München, Munich, Germany; 10 Institute for Clinical Diabetology, German Diabetes Center, Leibniz Center for Diabetes Research at Heinrich Heine University Düsseldorf, Düsseldorf, Germany; 11 Department of Endocrinology and Diabetology, Medical Faculty, University Hospital Düsseldorf, Heinrich Heine University Düsseldorf, Düsseldorf, Germany; 12 German Centre for Cardiovascular Research (DZHK), Partner Site Munich Heart Alliance, Munich, Germany

**Keywords:** DNA methylation, Type 2 diabetes, Glycemic traits, Diabetes progression, Gene expression

## Abstract

**Background:**

Type 2 diabetes (T2D) has been linked to changes in DNA methylation levels, which can, in turn, alter transcriptional activity. However, most studies for epigenome-wide associations between T2D and DNA methylation comes from cross-sectional design. Few large-scale investigations have explored these associations longitudinally over multiple time-points.

**Methods:**

In this longitudinal study, we examined data from the Cooperative Health Research in the Region of Augsburg (KORA) F4 and FF4 studies, conducted approximately seven years apart. Leucocyte DNA methylation was assessed using the Illumina EPIC and 450K arrays. Linear mixed-effects models were employed to identify significant associations between methylation sites and diabetes status, as well as with fasting plasma glucose (FPG), hemoglobin A1c (HbA1c), homoeostasis model assessment of beta cell function (HOMA-B), and homoeostasis model assessment of insulin resistance (HOMA-IR). Interaction effects between diabetes status and follow-up time were also examined. Additionally, we explored CpG sites associated with persistent prediabetes or T2D, as well as the progression from normal glucose tolerance (NGT) to prediabetes or T2D. Finally, we assessed the associations between the identified CpG sites and their corresponding gene expression levels.

**Results:**

A total of 3,501 observations from 2,556 participants, with methylation measured at least once across two visits, were included in the analyses. We identified 64 sites associated with T2D including 15 novel sites as well as known associations like those with the thioredoxin-interacting protein (*TXNIP*) and ATP-binding cassette sub-family G member 1 (*ABCG1*) genes. Of these, eight CpG sites exhibited different rates of annual methylation change between the NGT and T2D groups, and seven CpG sites were linked to the progression from NGT to prediabetes or T2D, including those annotated to mannosidase alpha class 2a member 2 (*MAN2A2*) and carnitine palmitoyl transferase 1 A (*CPT1A*). Longitudinal analysis revealed significant associations between methylation and FPG at 128 sites, HbA1c at 41 sites, and HOMA-IR at 57 sites. Additionally, we identified 104 CpG-transcript pairs in whole blood, comprising 40 unique CpG sites and 96 unique gene transcripts.

**Conclusions:**

Our study identified novel differentially methylated loci linked to T2D as well as to changes in diabetes status through a longitudinal approach. We report CpG sites with different rates of annual methylation change and demonstrate that DNA methylation associated with T2D is linked to following transcriptional differences. These findings provide new insights into the molecular mechanisms of diabetes development.

**Graphical abstract:**

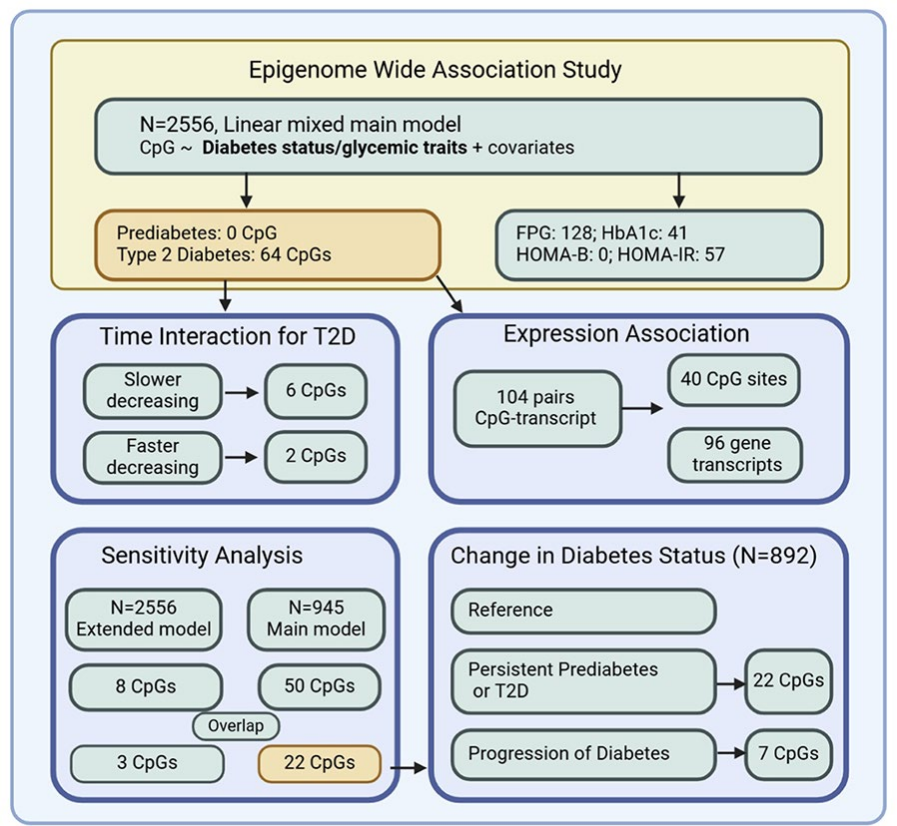

**Supplementary Information:**

The online version contains supplementary material available at 10.1186/s12933-024-02558-8.

## Background

Type 2 diabetes (T2D) is a major public health concern, characterized by chronic hyperglycemia. The prevalence of T2D is rising rapidly worldwide, projected to affect 783 million adults by 2045 [1]. Individuals with T2D are at risk of developing severe and life-threatening complications, leading to increased medical needs and reduced quality of life. Despite extensive research on T2D pathophysiology, the underlying mechanisms are not yet fully elucidated. Epigenetic modifications, especially DNA methylation—where methyl groups are added to DNA molecules affecting gene expression without altering the DNA sequence—are emerging as crucial links between genetic, environmental, and lifestyle factors in T2D development and progression [2–5]. Identification of novel biomarkers linked to T2D and early glucose disturbances can enhance our understanding of the disease’s etiology and improve prevention and prediction strategies [6, 7].

Advances in methylation technology have facilitated the simultaneous measurement of numerous cytosine-phosphate-guanine (CpG) dinucleotide sites, leading to the identification of various CpG sites associated with prevalent T2D and glycemic traits in cross-sectional epigenome-wide association studies (EWAS) [8–11]. Recent comprehensive analyses, including a systematic review of 32 studies, have summarized evidence linking DNA methylation patterns to T2D pathophysiology, utilizing samples from blood, pancreatic islet, adipose tissue, liver, spermatozoa and skeletal muscle [12]. Additionally, a study involving over 18,000 Scottish individuals examined the relationship between blood DNA methylation and the prevalence and incidence of multiple diseases, including T2D [13]. Furthermore, genome-wide DNA methylation changes in early life, particularly among offspring exposed to gestational diabetes, have been proposed as a potential mechanism that increase the risk of obesity, glucose intolerance, and T2D [14–16].

Previous studies have been cross-sectional, limiting insights into temporality. Methylation changes may either be part of the causal pathway to disease or serve as non-causal biomarkers [17, 18]. Considering the fluctuating nature of glucose and insulin metabolism prior to T2D development, it is essential to understand the evolution of methylation patterns in the progression from normal glucose tolerance (NGT) to prediabetes and T2D. For instance, maternal glycemia during pregnancy has been linked to longitudinal variations in blood DNA methylation at the fibronectin type III and spry domain containing 1 like (*FSD1L*) loci from birth to age five [19]. In addition, a cross-lagged analysis of twin samples in China demonstrated bidirectional associations between DNA methylation and T2D or glycemic traits, with significant paths from T2D influencing subsequent DNA methylation and vice versa [20]. In summary, few studies have examined longitudinal changes in methylation across multiple time points and existing longitudinal research often focuses on specific individuals or ancestries with small sample sizes. In our study, we aimed to investigate the association between DNA methylation and diabetes status, as well as four related traits—fasting plasma glucose (FPG), hemoglobin A1c (HbA1c), homoeostasis model assessment of insulin resistance (HOMA-IR) and homoeostasis model assessment of beta-cell function (HOMA-B)—within a longitudinal, population-based cohort comprising 2,556 individuals, utilizing up to two repeated measurements of DNA methylation as well as glucose- and insulin-related traits.

**Fig. 1:**
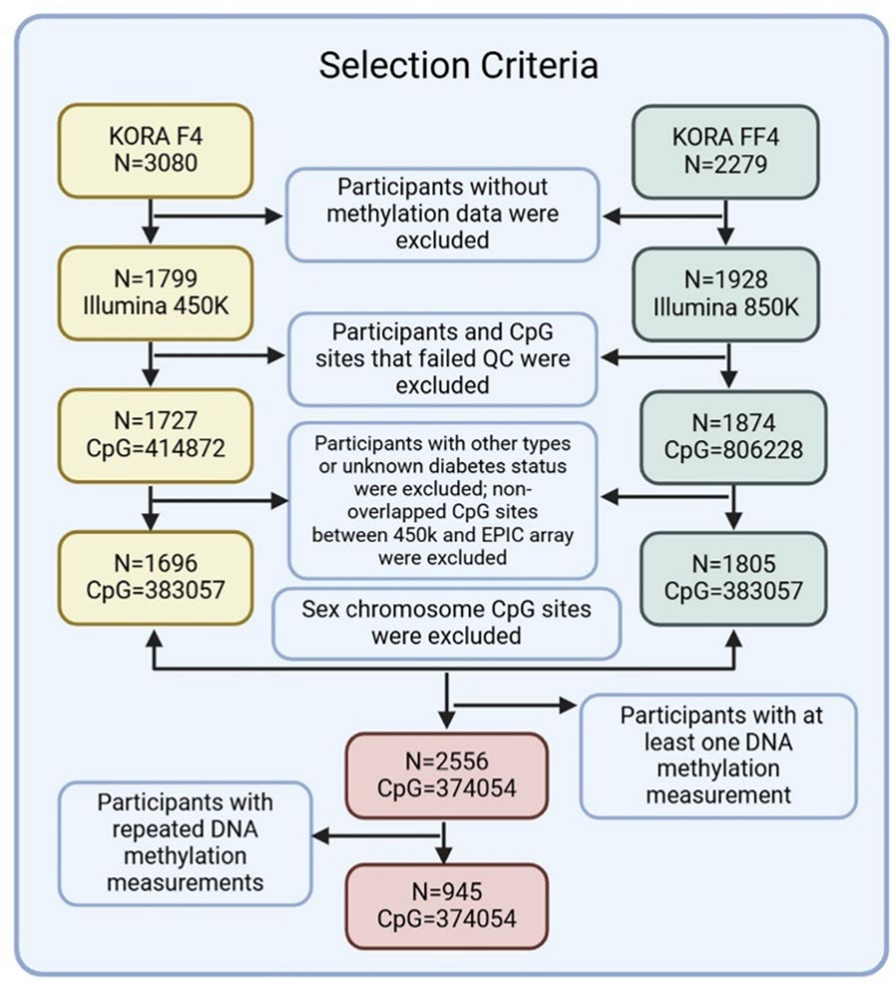
Illustration of the selection criteria for study participants and CpG sites included in the analysis

Illustration of the selection criteria for study participants and CpG sites included in the analysis.

## Methods

### Study population

This study used data from the Cooperative Health Research in the Region of Augsburg (KORA) F4 (2006–2008) and FF4 (2013–2014) studies, both follow-up studies of the KORA S4 study (1999–2001). Detailed information on the KORA cohort design, measurement, and data collection has been previously described [21]. In total, 3,501 observations from 2,556 participants in KORA F4 (1,696) and FF4 (1,805), with methylation data at least once across two visits, were included in the analysis. Of these participants, 945 participants (36.97%) had methylation patterns measured at both time points. Detailed information about the inclusion of study participants can be found in Additional file 1: Text S1.

### Measures of epigenome-wide DNA methylation and gene expression

In the KORA F4 study, genome-wide DNA methylation in whole blood was analysed using the Illumina 450K Infinium Methylation BeadChip (Illumina Inc., San Diego, CA, USA). For the KORA FF4 study, the Infinium MethylationEPIC BeadChip (Illumina Inc., San Diego, CA, USA) was used. DNA methylation was quantified on a scale of 0 to 1, with 1 signifying 100% methylation. We followed the general outline of the CPACOR preprocessing for quality control by using minfi2 package [22]. A total of 374,054 CpG sites were left for the analysis and detailed information about the quality control step and inclusion of CpG sites can be found in Fig. 1 and Additional file 1: Text S2 and Text S3. The proportions of white blood cell types (CD8T, CD4T, natural killer (NK) cells, B lymphocytes, monocytes and granulocytes) were estimated using the Reinius reference-based houseman algorithm implemented in the minfi package [23]. The algorithm is based on methylation values obtained from purified cell types in whole blood. These proportions were then utilized as covariates in the model to mitigate cell type confounding. The KORA F4 and FF4 datasets each included 470 and 448 non-negative control probes from the methylation arrays, respectively, with 430 probes overlapping. To address technical effects during the experiment, we conducted principal component analysis (PCA) on the overlapping probes. The resulting principal components (PCs) are believed to capture technical variability, and the first five control probe PCs, which accounted for 70% of the variance, were included as covariates in the model to eliminate technical biases. The generation and processing of the RNA-seq data of KORA FF4 are described in Additional file 1: Text S4. After quality control, the RNA-seq data were available for 1,543 individuals, with 10,671 gene counts retained for subsequent analysis.

### Measures of diabetes status

Previously known T2D was identified by self-report, validated by the responsible physician or medical chart review, or by self-reported current use of glucose-lowering medication. After an overnight fast of at least eight hours, participants without known diabetes underwent a standard 75 g oral glucose tolerance test (OGTT). NGT, prediabetes and newly diagnosed T2D were defined according to the 1999/2006 World health organization (WHO) criteria [24]. The specific cutoff values for the definition of T2D can be found in Additional file 1: Text S5. For this study, individuals with newly diagnosed T2D or previously known T2D were categorized as having T2D. Since this study involves longitudinal data, an individual’s diabetes status may change between time points. Abbreviations separated by a dash indicate diabetes status at baseline and at follow-up. For example, " prediabetes-T2D” refers to individuals who had prediabetes at baseline and had T2D at follow-up. FPG, HbA1c, HOMA-IR, and HOMA-B were assessed as described earlier [25].

### Statistical analysis

#### Epigenome wide association studies

We applied linear mixed-effects models with random participant-specific intercepts to examine the associations between DNA methylation (measured beta values ranging from 0 to 1) and diabetes status (NGT vs. prediabetes and T2D). The association between DNA methylation and diabetes status were identified by the epigenome wide association studies, adjusting for follow-up time (0 for baseline and the time difference to follow-up), age at baseline (years), sex (male, female), body mass index (BMI, kg/m^2^), smoking status (never, former, current), estimated cell types (monocytes, B Cells, CD4 T cells, CD8 T cells, and NK cells) and technical effects. An interaction term between sex and T2D was incorporated into the EWAS model to assess the differences in methylation levels between male and female individuals. We used the false discovery rate (FDR) (Benjamini–Hochberg method) to account for multiple testing. An association was considered statistically significant at a *p_*FDR value < 0.05. The same linear mixed effect model was applied to explore the association between DNA methylation and four continuous outcomes (FPG, HbA1c, HOMA-B and HOMA-IR), which were log-transformed to increase the conformity to normal distributions of residuals. Differentially methylated regions (DMRs) are genomic areas characterized by consistently differing DNA methylation levels across multiple adjacent CpG sites. Alongside the single-site position analysis, we utilized the comb-p function from the Enmix package (version 1.38.01) to identify diabetes-related DMRs. These were defined as groups of probes containing three or more positions within 1,000 base pairs of one another, with FDR-adjusted *p*-values of less than 0.05. To determine whether the identified diabetes-related CpG sites are also associated with other diseases or exhibit methylation changes in tissues beyond whole blood samples, we checked each significant CpG site in the EWAS Catalog [26].

#### Time interaction analysis

For CpG sites significantly associated with T2D in the main model, we examined their interaction effects between diabetes status and follow-up time. This interaction effect represents the difference in the rate of methylation change per year between individuals with and without T2D.

#### Sensitivity analysis

We conducted two sensitivity analyses to evaluate the robustness of our findings. First, we expanded our analysis by including additional confounding variables: parental history of diabetes (positive: at least one parent with diabetes; negative: both parents without diabetes; unknown), use of glucose-lowering medication (yes or no), HDL-cholesterol levels, triglyceride levels, and hypertension (yes or no). The detailed criteria used to assess or define these cofounders have been previously explained [27]. Second, we included only participants with repeated measures of both DNA methylation and glucose- and insulin-related traits, allowing for within-person comparisons over time (945 participants with 1,890 observations).

#### Association between DNA methylation and changing diabetes status

To investigate the association between DNA methylation and changing diabetes status over time, we categorized 945 participants individuals into 3 groups according to the diabetes status both at baseline and at follow-up: (i) 169 individuals who had either prediabetes or T2D at both time-points (prediabetes-prediabetes:67, T2D-T2D:102), (ii) 200 individuals who progressed from NGT to prediabetes or T2D, or from prediabetes to T2D (prediabetes-T2D:57, NGT-T2D:22, NGT-prediabetes:121), and (iii) 523 individuals who had NGT at both time-points (NGT-NGT: 523). We further excluded 53 individuals whose conditions improved over time, including those with T2D at baseline who had prediabetes or NGT at follow-up, and those with prediabetes at baseline who had NGT at follow-up (T2D-prediabetes:6, T2D-NGT:1, prediabetes-NGT:46) and finally 892 individuals left for the analysis. We focused on the previously identified overlapping significant CpG sites from the analysis of all individuals with methylation measured at least once across two visits (*N* = 2,556), as well as the subset with repeated DNA methylation measurements (*N* = 945).

#### Association between DNA methylation and gene expression

To investigate the relationship between the identified T2D-related CpG sites and gene expression, and to improve annotation, we analysed associations with gene expression probes within a 500 kb window surrounding the significant CpG sites. The MatrixEQTL (version 2.3) package was used to identify significant CpG-transcript associations. Linear models were adjusted for age, sex, measured white blood cell proportions (neutrophils, monocytes, basophils, and eosinophils) and technical variation with FDR correction for multiple testing.

#### Pathway analysis

To gain insights into potential biological processes relevant to diabetes or glycemic regulation, we performed gene pathway analysis using the GOmeth function from the missMethyl package (version 1.38.0). Pathways with an *p_*FDR < 0.05 were considered significant association.

## Results

### Characteristics of the study population

The analysis included 3,501 observations from 2,556 participants in the KORA F4 (1,696) and FF4 (1,805) studies. Table 1 presents the characteristics of all participants, while Additional file 1: Table S1 shows the characteristics of the 945 individuals with methylation measured at both time points. For all participants, the mean age was 61.0 years in F4 and 58.0 years in FF4. Among the 945 participants with repeated methylation measurements, the mean age was 57.0 years in F4 and 64.0 years in FF4. Due to differences in average age between the two cohorts, we included baseline age as a covariate in our linear mixed effects model to control for age-related variability. The mean BMI was 27.5 kg/m^2^ in F4 and 27.0 kg/m^2^ in FF4. Male participants comprised 48.8% of the F4 cohort and 48.1% of the FF4 cohort. Additionally, 14.5% of participants in F4 and 13.2% in FF4 had T2D, while 22.4% and 27.8%, respectively, had a parental history of diabetes.

**Table 1:** Characteristics of the study population

Characteristics	KORA F4				KORA FF4			
	All *N* = 1696	NGT *N* = 1113	Prediabetes *N* = 338	T2D *N* = 245	All *N* = 1805	NGT *N* = 1262	Prediabetes *N* = 304	T2D *N* = 239
Age (years)	61 (14)	58 (14)	65 (14)	67 (10)	58 (18)	54.5 (16)	63 (16)	68 (13.5)
Male (%)	828 (48.8%)	499 (44.8%)	184 (54.4%)	145 (59.2%)	868 (48.1%)	554 (43.9%)	172 (56.6%)	142 (59.4%)
BMI (kg/m^2^)	27.5 (5.8)	26.2(5.2)	29.3 (5.7)	30.7(6.7)	27.0 (6.2)	26.0 (5.4)	29.2 (5.2)	30.4 (7.2)
*Smoking*								
Never smoker	710 (41.9%)	460 (41.3%)	156 (46.2%)	94 (38.4%)	746 (41.3%)	522 (41.4%)	118 (38.8%)	106 (44.4%)
Former smoker	737 (43.5%)	462 (41.5%)	156 (46.2%)	119 (48.6%)	766 (42.4%)	517 (41.0%)	138 (45.4%)	111 (46.4%)
Current smoker	247 (14.6%)	189 (17.0%)	26 (7.7%)	32 (13.1%)	293 (16.2%)	223 (17.7%)	48 (15.8%)	22 (9.2%)
Hypertension (%)	772 (45.5%)	377 (33.9%)	198 (58.6%)	197 (80.4%)	646 (35.8%)	317 (25.1%)	159 (52.3%)	170 (71.1%)
Fasting glucose	5.4 (0.9)	5.2 (0.6)	5.8 (0.9)	6.9 (1.9)	5.4 (0.9)	5.2 (0.6)	6.1 (0.8)	7.2 (2.0)
HOMA-IR	2.2 (1.8)	1.9 (1.3)	3.1 (2.5)	5.1 (4.0)	2.1 (1.9)	1.8 (1.4)	3.5 (2.2)	4.8 (4.2)
HOMA-beta	102.0 (65.7)	101.0 (62.7)	110.0 (79.0)	93.5 (97.4)	94.8 (65.5)	93.1 (61.0)	110.0 (87.7)	102. (70.3)
HbA1c	37.0 (6.0)	36.0 (5.0)	38.5 (5.0)	46.0 (12.0)	36.0 (6.0)	34.0 (5.0)	38.0 (5.0)	45.0 (10.8)
HDL-cholesterol	1.4 (0.5)	1.5 (0.5)	1.3 (0.5)	1.2 (0.4)	1.6 (0.7)	1.7 (0.7)	1.5 (0.6)	1.4 (0.5)
Triglycerides	1.3 (0.9)	1.1 (0.8)	1.5 (1.0)	1.7 (1.2)	1.2 (0.8)	1.1 (0.7)	1.5 (1.0)	1.6 (1.2)
Medication	128.0 (7.6%)	0 (0%)	0 (0%)	128 (52.2%)	133 (7.4%)	0 (0%)	0 (0%)	133 (55.6%)
*Parental history*								
Yes	380 (22.4%)	239 (21.5%)	71 (21.0%)	70 (28.6%)	501 (27.8%)	314 (24.9%)	94 (30.9%)	93 (38.9%)
No	773 (45.6%)	582 (52.3%)	135 (39.9%)	56 (22.9%)	1131 (62.7%)	844 (66.9%)	177 (58.2%)	110 (46.0%)
Unknown	254 (15.0%)	159 (14.3%)	53 (15.7%)	42 (17.1%)	173 (9.6%)	104 (8.2%)	33 (10.9%)	36 (15.1%)

Data are median (IQR) for continuous variables and n (%) for categorical variables. The unit for both fasting glucose and HbA1c is mmol/mol. The unit for both HDL-cholesterol and triglycerides is mmol/l. Medication means the glucose-lowering medication

### Longitudinal association between DNA methylation and diabetes status

**Fig. 2:**
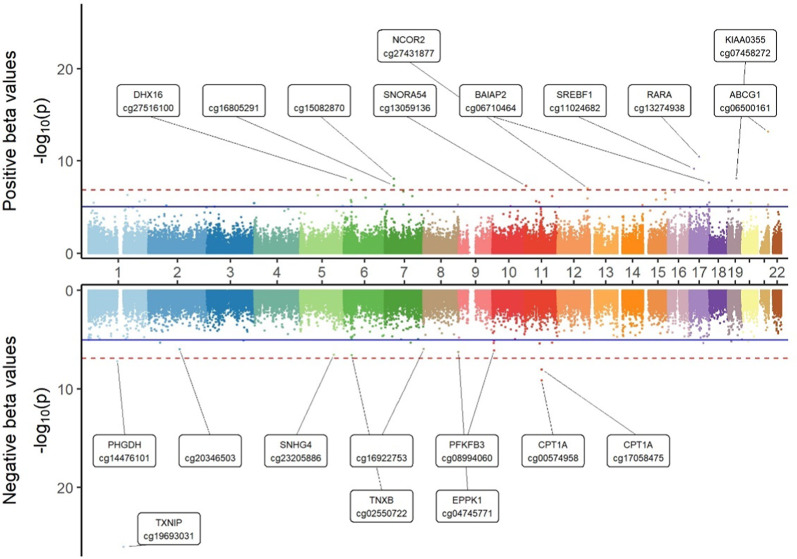
Miami plot illustrating EWAS results associated with T2D. The x axis indicates the chromosome location, and the y-axis represents the −log10 (p-value). The Bonferroni threshold of 1.34×10−7 is marked by a red dashed line, while the Benjamini–Hochberg (FDR) threshold (*p_*FDR < 0.05) is indicated by a blue solid line. The upper side represents the positive estimates, and the lower side represents the negative estimates

An EWAS was conducted to identify differences in DNA methylation among individuals with NGT, prediabetes and T2D using linear mixed effect models with individual-specific random intercepts in a longitudinal study. Among the 374,054 CpG sites examined, none showed a significant association with prediabetes, while 64 sites (annotated to 47 unique genes) exhibited significant associations with T2D, with 21 sites being hypomethylated and 43 sites being hypermethylated compared to individuals with NGT. Diabetes-by-sex interaction analysis revealed no significant differences between men and women. The Miami plot (Fig. 2) illustrates the distribution of CpG sites associated with T2D. Table 2 provides a summary of the 15 most significant CpG sites, while Additional file 2: Table S1 lists all significant CpG sites linked to T2D. Notably, cg19693031, annotated to thioredoxin-interacting protein (*TXNIP*), emerged as the most significant CpG site (*p* value: 9.51 × 10^− 27^) and demonstrated the most significant effect size in our analysis (− 2.92%). The results confirm 49 previously reported cross-sectionally associated gene loci, including those annotated to *TXNIP*, ATP-binding cassette sub-family G member 1 (*ABCG1*), carnitine palmitoyl transferase 1 A (*CPT1A*), and sterol regulatory element-binding transcription factor 1 (*SREBF1*). Importantly, the effect direction of these associations in this longitudinal study was consistent with those of the cross-sectional results for all 49 known sites listed in the EWAS catalogue [26]. Additionally, 15 CpG sites annotated to 10 unique genes were identified as novel associations, including cg02550722 annotated to tenascin XB (*TNXB*), cg04745771 annotated to epiplakin 1 (*EPPK1*), cg23661483 annotated to ilvb acetolactate synthase like (*ILVBL*), cg13947735 annotated to UDP-glcnac: betagal beta-1,3-n-acetylglucosaminyltransferase like 1 (*B3GNTL1*), cg15418499 annotated to interleukin-18 (*IL18*), cg14172849 annotated to X-ray repair cross complementing 3 (*XRCC3*), cg20661985 annotated to open reading frame 3 encoded at human chromosome 20 (*C20orf3*). The DMR analysis identified 44 significant regions associated with 36 unique genes. This analysis confirmed 7 genes previously identified in the single position analysis and uncovered 29 novel genes linked to T2D, such as valyl-tRNA synthetase (*VARS*), or solute carrier family 1 member 5 (*SLC1A5*). Detailed information related to the DMR analysis is available in Additional file 2: Table S2. The identified T2D-related CpG sites are also linked to other diseases, including metabolic syndrome and cardiovascular diseases, and show methylation changes in specific tissues, such as the liver. For detailed information, please refer to Additional file 2: Table S3.

Miami plot illustrating EWAS results associated with T2D. The x axis indicates the chromosome location, and the y-axis represents the − log10 (*p*-value). The Bonferroni threshold of 1.34 × 10^− 7^ is marked by a red dashed line, while the Benjamini–Hochberg (FDR) threshold (*p_*FDR < 0.05) is indicated by a blue solid line. The upper side represents the positive estimates, and the lower side represents the negative estimates.

**Table 2:** Summary of top 15 significant CpG sites associated with T2D

Probe	Delta beta (%)	*p* value	*p*_FDR	CHR	Gene	MAPINFO	Gene_group
cg19693031	− 2.92	9.51E−27	3.55E−21	1	*TXNIP*	145,441,552	3’UTR
cg06500161	1.22	6.69E−14	1.25E−08	21	*ABCG1*	43,656,587	Body
cg13274938	0.91	3.30E−11	4.12E−06	17	*RARA*	38,493,822	Body
cg11024682	0.95	6.78E−10	5.43E−05	17	*SREBF1*	17,730,094	Body
cg00574958	− 0.73	7.26E−10	5.43E−05	11	*CPT1A*	68,607,622	5’UTR
cg07458272	1.02	7.75E−09	4.45E−04	19	*KIAA0355*	34,744,396	TSS1500
cg15082870	0.91	8.44E−09	4.45E−04	7	*#*	36,022,841	#
cg17058475	− 1.06	9.53E−09	4.45E−04	11	*CPT1A*	68,607,737	5’UTR
cg27516100	0.83	1.11E−08	4.64E−04	6	*DHX16*	30,624,520	Body
cg06710464	0.94	2.24E−08	8.38E−04	17	*BAIAP2*	79,047,695	Body
cg16805291	1.15	4.70E−08	1.58E−03	7	*#*	36,022,575	#
cg13059136	1.06	5.08E−08	1.58E−03	11	*SNORA54*	2,986,541	TSS1500
cg14476101	− 1.46	6.48E−08	1.86E−03	1	*PHGDH*	120,255,992	Body
cg27431877	0.60	8.83E−08	2.35E−03	12	*NCOR2*	124,911,924	Body
cg01676795	1.18	2.02E−07	5.04E−03	7	*POR*	75,586,348	Body

Probe: Unique identifier from the Illumina CG database; Delta Beta: Mean methylation difference between T2D and NGT; *p_*FDR: Benjamini-Hochberg corrected *p* value (FDR); CHR: Chromosome; Gene: Target gene name from the UCSC database (# indicates no annotated gene); MAPINFO: Chromosomal coordinates of the CpG (Build 37); Gene_Group: Gene region feature category describing the CpG position from UCSC

### Longitudinal association between DNA methylation and glycemic traits

**Fig. 3:**
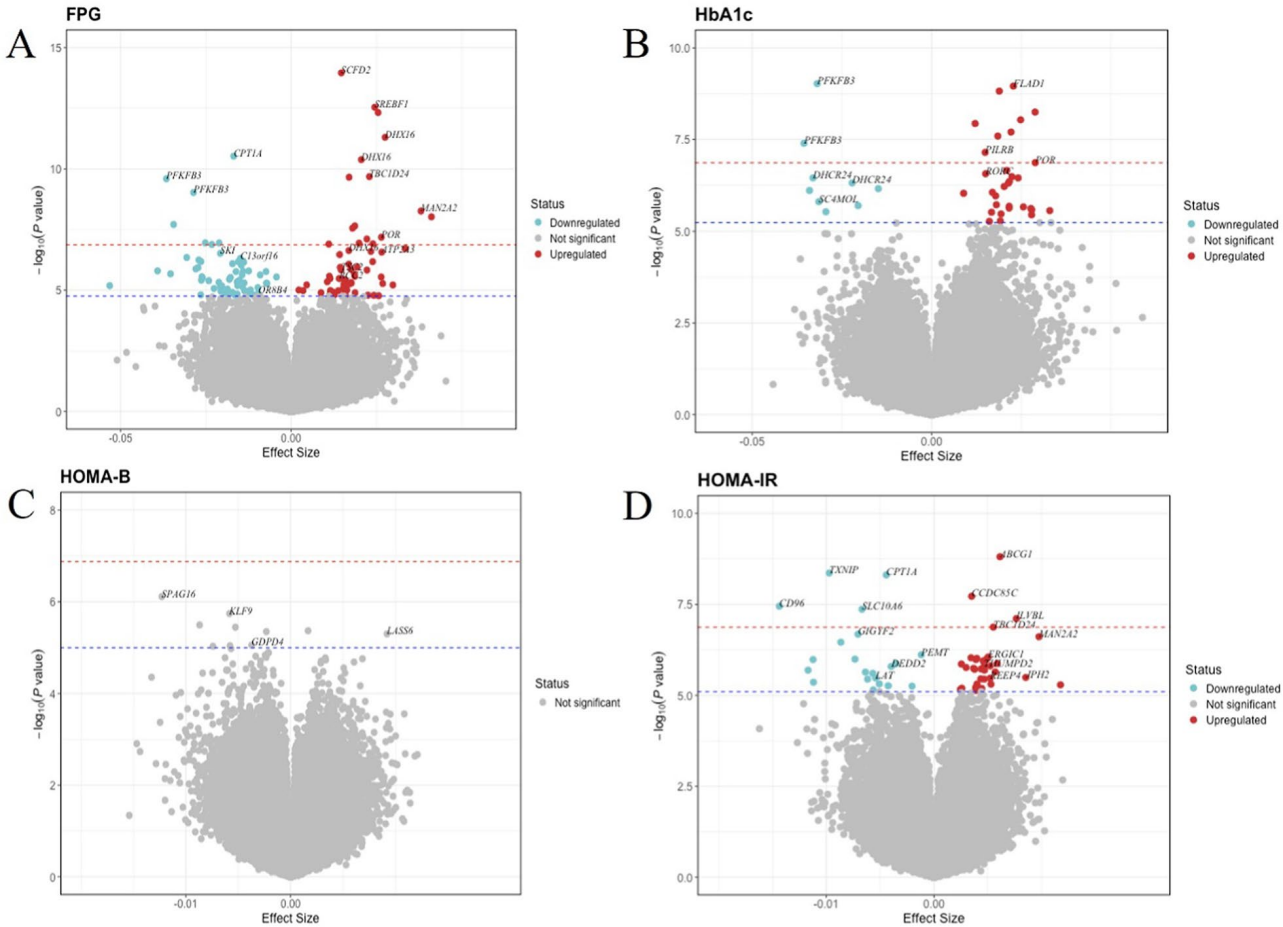
Volcano plots illustrating the results for glycemic traits. The x axis indicates the effect size, and the y-axis represents the −log10 (p-value). The Bonferroni threshold of p=1.34×10−7 is marked by a red dashed line, while the Benjamini–Hochberg (FDR) threshold (*p_*FDR < 0.05) is indicated by a blue dashed line. (A) Volcano plot for FPG. (B) Volcano plot for HbA1c. (C) Volcano plot for HOMA-B. (D) Volcano plot for HOMA-IR.

The same EWAS model was employed to evaluate the longitudinal association between DNA methylation and four glycemic traits: FPG, HbA1c, HOMA-B, and HOMA-IR. Out of the 374,054 CpG sites examined, 128 were associated with FPG, 41 with HbA1c, none with HOMA-B, and 57 with HOMA-IR. Notably, two CpG sites, cg19693031 (*TXNIP*) and cg06500161 (*ABCG1*), were associated with FPG, HbA1c, HOMA-IR, and T2D. The glycemic trait analysis identified an additional 161 unique CpG sites distinct from those associated with T2D, bringing the total number of unique CpG sites linked to both T2D and glycemic traits to 225. Volcano plots (Fig. 3) illustrate the direction of association of the significant CpG sites related to glycemic traits. Additional file 2: Tables S4-6 provide detailed information on all significant CpG sites linked to glycemic traits.

Volcano plots illustrating the results for glycemic traits. The x axis indicates the effect size, and the y-axis represents the − log10 (*p*-value). The Bonferroni threshold of *p* = 1.34 × 10^− 7^ is marked by a red dashed line, while the Benjamini–Hochberg (FDR) threshold (*p_*FDR < 0.05) is indicated by a blue dashed line. (A) Volcano plot for FPG. (B) Volcano plot for HbA1c. (C) Volcano plot for HOMA-B. (D) Volcano plot for HOMA-IR.

### Interaction between diabetes status and follow-up time

**Fig. 4:**
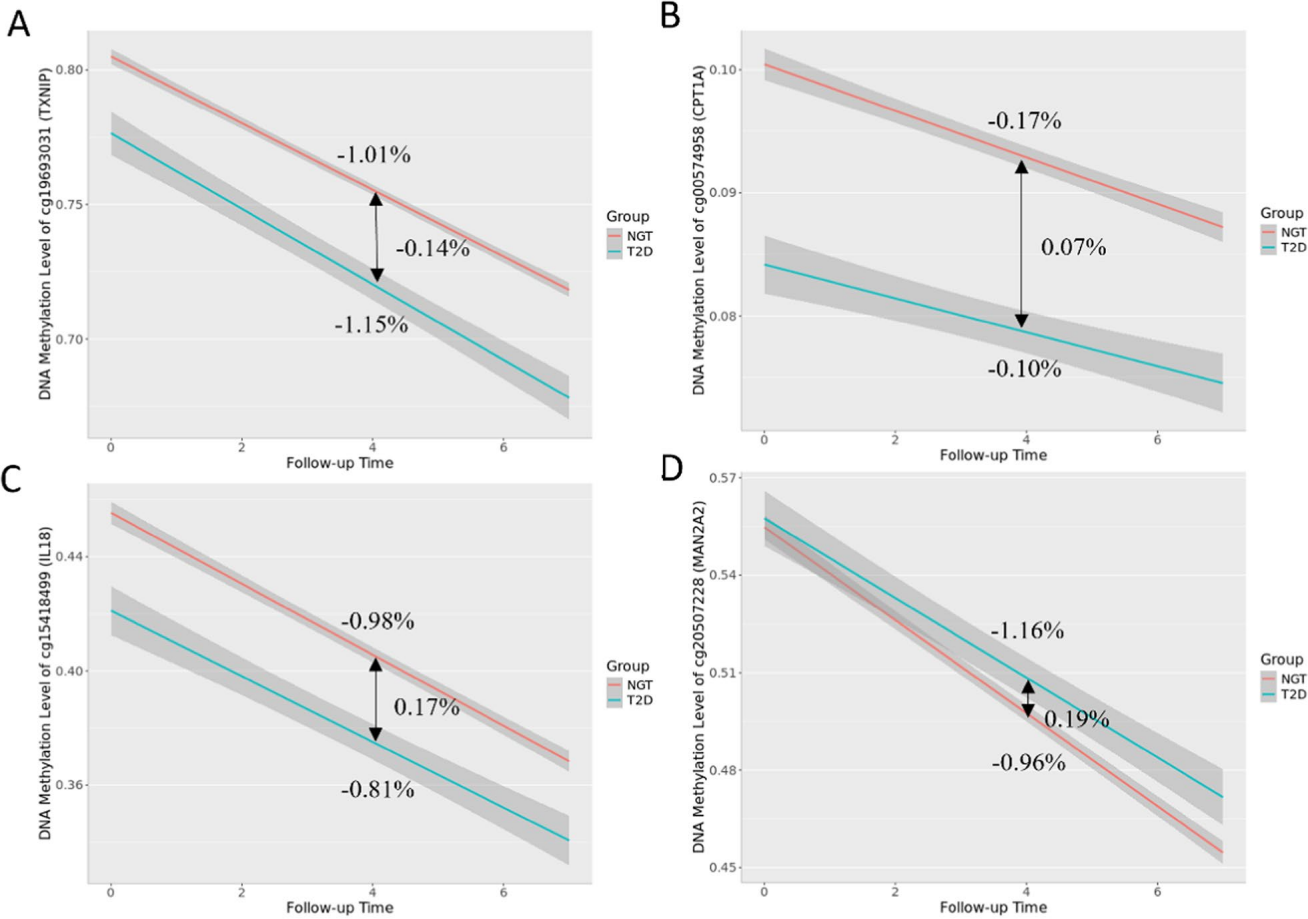
Line plots illustrating the rate of methylation change over time for the NGT and T2D groups. The red and blue line represents the individuals with NGT and T2D, respectively. (A) cg19693031 (*TXNIP*); (B) cg00574958 (*CPT1A*); (C) cg15418499 (*IL18*); (D) cg20507228 (*MAN2A2*).

We focused on the 64 CpG sites that showed significant associations with T2D in the main model and added an interaction term between T2D and follow-up time to the model. This estimate indicates the difference of the methylation change rates between individuals with T2D and NGT. Eight CpG sites were considered significant (*p_*FDR value < 0.05). All 8 CpG sites showed a decrease in methylation levels over time. Two CpG sites, cg20346503 and cg19693031 (annotated to *TXNIP*), exhibited a steeper decline in methylation for individuals with T2D compared to those with NGT, with methylation rates of -1.22% and − 1.01% for NGT, versus − 1.31% and − 1.15% for T2D, respectively. In contrast, six CpG sites (cg10442325, cg15418499 annotated to *IL18*, cg20507228, annotated to *MAN2A2*, cg04334723 annotated to calreticulin (*CALR*), cg20661985 and cg00574958 annotated to *CPT1A*) exhibited a slower decrease in methylation change over time for individuals with T2D compared to those with NGT. For instance, the slope for *CPT1A* was − 0.17% for NGT versus − 0.10% for T2D. Furthermore, our analysis demonstrated that there are no interaction effects among male and female participants. Table 3 and Additional file 2: Table S7 provide summary information about the CpG sites which showed interaction effects with follow-up time. Figure 3 and Additional file 1: Fig.S1 illustrate the rate of methylation change over time for the NGT and T2D groups (Fig. 4).

**Table 3:** Summary of 8 significant CpG sites with different methylation change rates over time for individuals with T2D compared to those with NGT

Probe	Estimate1 (%)	Estimate2 (%)	Estimate3 (%)	*p*value	*p*_FDR	Gene	Gene_group
cg10442325	− 0.86	− 0.71	0.14	3.81E-05	0.002	*#*	#
cg15418499	− 0.98	− 0.81	0.17	8.19E-04	0.023	*IL18*	5’UTR
cg20507228	− 1.16	− 0.96	0.19	1.10E-03	0.023	*MAN2A2*	Body
cg04334723	− 0.79	− 0.68	0.10	2.15E-03	0.031	*CALR*	Body
cg20346503	− 1.22	− 1.31	− 0.09	2.48E-03	0.031	*#*	#
cg19693031	− 1.01	− 1.15	− 0.14	3.40E-03	0.031	*TXNIP*	3’UTR
cg20661985	− 1.39	− 1.25	0.13	3.46E-03	0.031	*C20orf3*	Body
cg00574958	− 0.17	− 0.10	0.07	6.02E-03	0.048	*CPT1A*	5’UTR

Probe: Unique identifier from the Illumina CG database; Estimate1: the estimate of follow-up time indicating the methylation change rate per year for individuals with NGT; Estimate2: the methylation change rate per year for individuals with T2D by adding Estimate1 and Estimate3; Esimate3: the estimate of the interaction term between diabetes and follow-up time indicating the difference of methylation change rates between NGT and T2D; *p_*FDR: Benjamini-Hochberg corrected *p* value; Gene: Target gene name from the UCSC database. Gene_Group: Gene region feature category describing the CpG position from UCSC

Line plots illustrating the rate of methylation change over time for the NGT and T2D groups. The red and blue line represents the individuals with NGT and T2D, respectively. (A) cg19693031 (*TXNIP*); (B) cg00574958 (*CPT1A*); (C) cg15418499 (*IL18*); (D) cg20507228 (*MAN2A2*).

### Sensitivity analysis

In our sensitivity analysis, we further adjusted for medication use, parental history of diabetes, HDL-cholesterol, triglycerides, and hypertension as the extended model. Among the 374,054 CpG sites examined, 8 sites were associated with T2D. Of these, 3 CpG sites remained significant and consistent with our main analysis results. These include cg19693031 annotated to *TXNIP* (effect size: -1.83%, *p* value: 1.31 × 10^− 7^), cg06500161 annotated to *ABCG1* (effect size: 0.20%, *p* value: 1.41 × 10^− 7^), and cg13274938 annotated to retinoic acid receptor alpha (*RARA*) (effect size: 0.92%, *p* value: 9.93 × 10^− 7^).

**Fig. 5:**
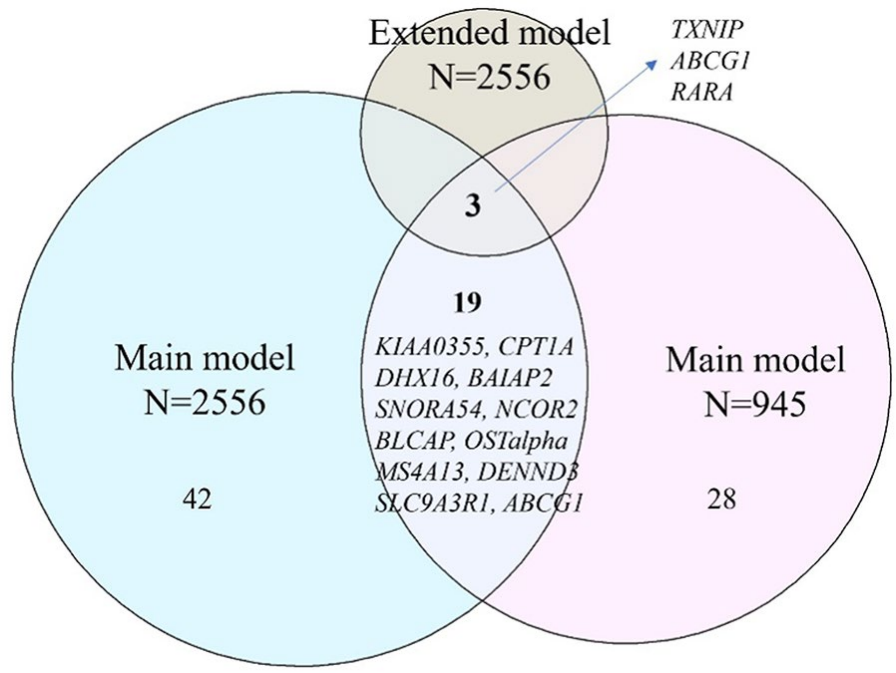
Venn diagram illustrating the overlap of CpG sites (with annotated gene names) in the sensitivity analysis. The light cyan colour represents the number of significant CpG sites associated with T2D in the main analysis with all individuals. The greyish-yellow colour represents the number of significant CpG sites associated with T2D in the extended models with all individuals. The light pink colour represents the number of significant CpG sites associated with T2D from individuals with repeated methylation measurements at two time points.

We also conducted a sensitivity analysis on a subset of 945 individuals with repeated methylation measurements. Among the 374,054 CpG sites examined, 50 CpG sites were associated with T2D and the associations for 22 of these sites, including *TXNIP*, *ABCG1* and *RARA*, remained robust. The correlation coefficients of estimates and *p* values between the full cohort (*N* = 2,556) and the repeated methylation measurement subset (*N* = 945) was strong (*r* = 0.78) and moderate (*r* = 0.45), respectively. The Venn diagram (Fig. 5) illustrates the overlap of CpG sites across different datasets, while the Manhattan plots (Additional file 1: Fig. S2) and Additional file 2: Tables S8-9 present results from the extended model and the subset analysis.

Venn diagram illustrating the overlap of CpG sites (with annotated gene names) in the sensitivity analysis. The light cyan colour represents the number of significant CpG sites associated with T2D in the main analysis with all individuals. The greyish-yellow colour represents the number of significant CpG sites associated with T2D in the extended models with all individuals. The light pink colour represents the number of significant CpG sites associated with T2D from individuals with repeated methylation measurements at two time points.

### Association between DNA methylation and changing diabetes status over time

**Fig. 6:**
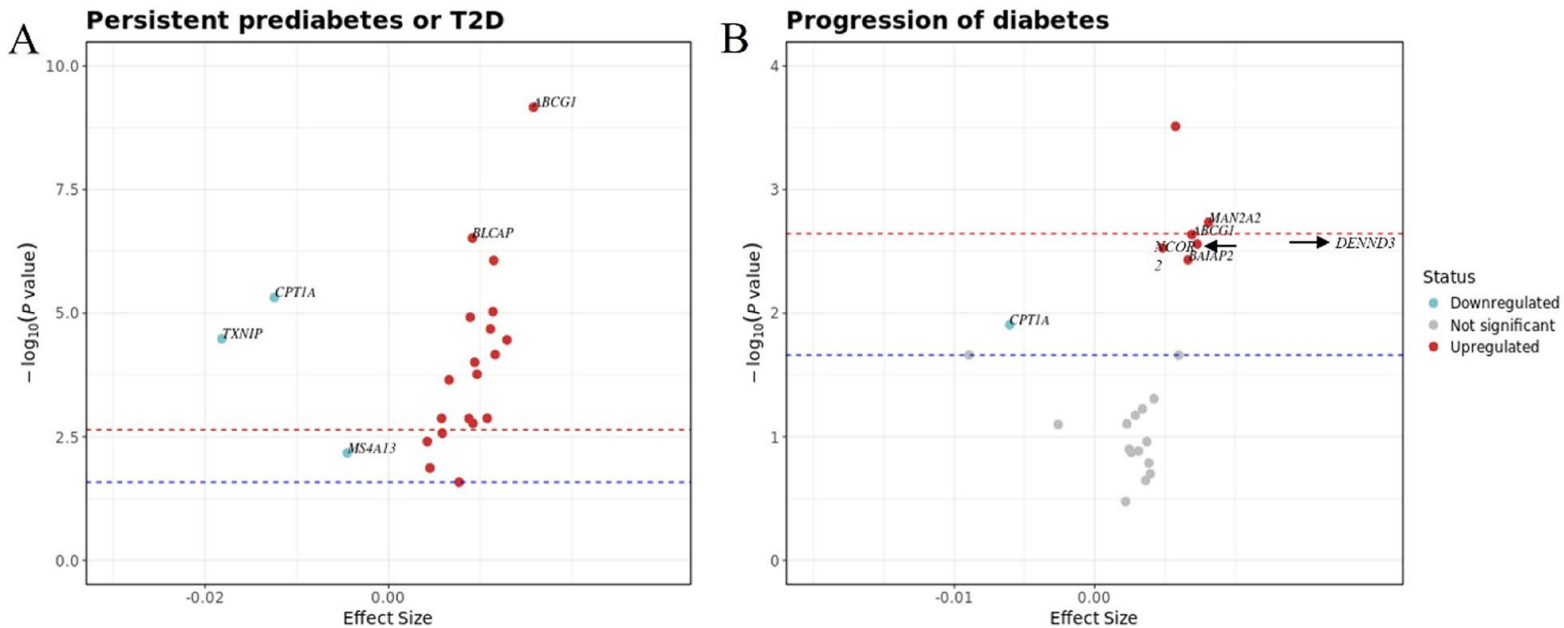
Volcano plots illustrating the association between DNA methylation and changing diabetes status over time. The x axis indicates the effect size, and the y-axis represents the −log10 (p-value). The Bonferroni threshold of 2.27×10−3 is marked by a red dashed line, while the Benjamini–Hochberg (FDR) threshold (*p_*FDR < 0.05) is indicated by a blue dashed line. (A) Volcano plot for the persistent prediabetes or T2D. (B) Volcano plot for the progression of diabetes.

The analysis focused on the 22 CpG sites that were associated with T2D in both the full cohort (*N* = 2,556) and the subset cohort (*N* = 945). Among these 22 CpG sites, all showed significant associations with persistent prediabetes or T2D at both timepoints, while 7 showed significant associations with progression of diabetes status either from NGT to prediabetes or T2D or from prediabetes to T2D. Notably, these 7 CpG sites, including cg23436042, cg11183227 annotated to *MAN2A2*, cg06500161 annotated to *ABCG1*, cg08788930 annotated to DENN domain-containing protein 3 (*DENND3*), cg11311053 annotated to nuclear receptor corepressor 2 (*NCOR2*), cg06710464 annotated to BAR/IMD domain containing adaptor protein 2 (*BAIAP2*), and cg17058475 annotated to *CPT1A*, demonstrated associations with both persistent and progressed diabetes status. Volcano plots (Fig. 6) illustrate the direction of associations of these significant CpG sites, while the Venn plot (Additional file 1: Fig.S3) shows the overlap of CpG sites across different groups. Additional file 2: Tables S10-11 provide summaries of the significant CpG sites linked to persistent and progressed diabetes status, respectively.

Volcano plots illustrating the association between DNA methylation and changing diabetes status over time. The x axis indicates the effect size, and the y-axis represents the − log10 (*p*-value). The Bonferroni threshold of 2.27 × 10^− 3^ is marked by a red dashed line, while the Benjamini–Hochberg (FDR) threshold (*p_*FDR < 0.05) is indicated by a blue dashed line. (A) Volcano plot for the persistent prediabetes or T2D. (B) Volcano plot for the progression of diabetes.

### Association between DNA methylation and gene expression

Focusing on the 64 significant T2D-related CpG sites, we identified 104 CpG-transcript pairs in whole blood, involving 40 unique CpG sites and 96 unique gene transcripts. Of these, 48 pairs showed positive associations with an average effect size of 0.58, while 56 pairs showed negative associations with an average effect size of -1.02. For example, cg06500161 in *ABCG1* and cg06710464 in *BAIAP2* were negatively associated with their corresponding gene transcripts, while cg24704287 in latrophilin 1 (*LPHN1)* was positively associated with its corresponding gene transcript. Table 4 shows the top 10 significant associations; Additional file 2: Tables S12 summarizes the CpG-transcript associations.

**Table 4:** Top 10 associated CpG-transcript pairs

CpG	Gene	*p* value	FDR	Beta
cg06500161	*ABCG1*	1.17E−46	5.80E−44	− 4.76
cg06710464	*BAIAP2*	2.84E−41	7.04E−39	− 3.30
cg24704287	*LPHN1*	2.11E−29	3.49E−27	1.02
cg27243685	*ABCG1*	3.97E−28	4.91E−26	− 4.76
cg11024682	*SREBF1*	4.32E−26	4.28E−24	− 2.31
cg01676795	*POR*	3.23E−22	2.67E−20	− 1.18
cg06710464	*BAIAP2-AS1*	4.83E−22	3.41E−20	− 2.12
cg00851028	*TARBP1*	2.69E−15	1.66E−13	− 1.36
cg26340740	*MPEG1*	4.90E−12	2.69E−10	− 1.21
cg10691109	*COG5*	1.10E−11	5.48E−10	− 0.95

Statistically significant associations between metabolic measure-associated CpG sites and expression of cis-transcripts in whole blood (FDR-adjusted significance threshold *p* < 0.05). Gene: transcript ID; beta: coefficient between methylation and gene transcripts.

### Pathway analysis

In the pathway analysis of the 225 CpG sites associated with T2D and glycemic traits, no significant pathways were identified. The list of non-significant pathways is provided in Additional file 1: Fig.S5 and Additional file 2: Table S13.

## Discussion

This study employed longitudinal data with repeated measurements to explore the association between DNA methylation and diabetes status, as well as glycemic traits. We analysed 3,501 observations from 2,556 participants using linear mixed-effects models and identified 64 CpG sites associated with T2D. Notably, DNA methylation at 49 of these loci, including *TXNIP*, *ABCG1*, *CPT1A*, and *SREBF1*, exhibited consistent directional associations in our longitudinal analysis compared to previously reported cross-sectional studies [13, 28]. Importantly, our study revealed 15 novel CpG sites within 10 unique genes. Furthermore, we observed a distinct rate of methylation change for 8 CpG sites between the NGT and T2D groups, including those annotated to *IL18*, *MAN2A2*, *CALR*, *C20orf3* and *CPT1A*, which exhibited either faster or slower decreasing trends. Additionally, 7 CpG sites annotated to *MAN2A2*, *ABCG1*, *DENND3*, *NCOR2*, *BAIAP2* and *CPT1A* were linked to changes in diabetes status. Moreover, we identified 104 associations between identified significant T2D-related CpG sites and their corresponding gene expression levels.

The 64 significant sites that differ between individuals with T2D and NGT in our longitudinal study are annotated to 49 unique genomic loci. *TXNIP* (1 site) has consistently emerged as the most significant gene associated with T2D in previous EWAS studies [29] due to its role in regulating pancreatic β-cells production and survival [30] and has arisen as a novel potential therapeutic target in diabetes mellitus and its complications [31]. *RARA* (1 site), the gene encoding retinoic acid receptor alpha, is a well-known gene linked to cigarette smoking [32]. *FoxK2* (1 site), a major target of insulin signalling, plays a critical role in apoptosis, metabolism, and mitochondrial function [33] and could regulate aerobic glycolysis [34]. Dyslipidaemia and diabetes are closely related, and epigenome-wide approaches have identified differential methylation of genes known to have a key role in lipid metabolism and lipid traits, particularly *CPT1A*, *ABCG1*, *SREBF1* [35–38]. *ABCG1* (2 sites) is crucial for cholesterol efflux [39], and cg06500161 within *ABCG1* has been reported to mediate the association between statins and risk of T2D [40]. *CPT1A* (2 sites) is associated with an increased risk of gestational diabetes mellitus (GDM) [41]. And multi-tissue epigenetic analysis has revealed distinct associations between the *CPT1A* locus and insulin resistance [42]. Risk group stratification based on cg11024682 (*SREBF1*) was reported to be valuable for personalized T2D risk prediction [43, 44]. Our study found that after controlling for lipid levels in extended models, the associations at the *ABCG1* loci remained robust. In contrast, the associations for *CPT1A* and *SREBF1* were not maintained, suggesting that these associations might be driven by alterations in lipid metabolism.

Our study identified 15 novel CpG sites annotated to 10 unique genes, including *TNXB*, *EPPK1*, *ILVBL*, *B3GNTL1*, *IL18*, *XRCC3*, *C20orf3*. Hypomethylation of *TNXB* gene and differential expression of *EPPK1* protein in the placenta has been reported to be associated with GDM [45, 46]. In a mouse model of diabetes, *ILVBL* has been reported to be involved in the formation of increased dimethylglyoxal, which induces oxidative stress and disrupts the blood-brain barrier, potentially leading to neurological complications in diabetes [47]. *B3GNTL1* was identified as part of a trans-omics biomarker for diabetic kidney disease in diabetic patients [48]. *XRCC3*, a DNA repair gene, has been significantly associated with T2D and diabetic nephropathy in a Turkish population [49]. *C20orf3*, an adipocyte plasma membrane-associated protein, was found to be down-regulated in omental adipose tissues from individuals with GDM [50]. Previous studies have shown that blood methylation patterns in adipose tissue change after bariatric surgery, particularly in genes related to immune system, suggesting that blood DNA methylation reflects the inflammatory state of adipose tissue post-surgery [51]. In our study, we also found that the identified T2D-related CpG sites are also showed methylation changes in specific tissues, such as the liver, by comparing them to the EWAS catalog.

Prolonged disturbances in glucose metabolism are often observed before diabetes diagnosis. Diagnostic tools like FPG and HbA1c are critical for identifying diabetes, underscoring the significance of investigating their effects on DNA methylation. A systematic review and meta-analysis revealed that high HOMA-IR values were positively associated with an increase in risk of T2D [52]. Previous studies have explored the association between DNA methylation changes and hyperglycaemia exposure using the longitudinal D.E.S.I.R. cohort over a six-year period but did not find significant results [53]. Notably, in our study, two CpG sites, cg19693031 (*TXNIP*) and cg06500161 (*ABCG1*), were simultaneously associated with FPG, HbA1c, HOMA-IR, and T2D. These findings highlight the link between glycemic parameters, insulin resistance and DNA methylation, suggesting that alterations at specific CpG sites could serve as biomarkers for glycaemic control and diabetes risk prediction.

DNA methylation is the most studied epigenetic regulator related to environmental exposures. Various environmental triggers, including chemical exposures and complex disease conditions, can lead to global or site-specific DNA methylation changes. This regulation allows for immediate environmental adaptations, potentially affecting transcription factor binding and gene expression. Importantly, we observed that the rate of methylation change varied across diabetes groups. Eight CpG sites, annotated to six unique genes—*IL18*, *MAN2A2*, *CALR*, *TXNIP*, *C20orf03*, and *CPT1A*—all showed decreasing methylation values over time. Low blood *TXNIP* DNA methylation has been linked to increased glucose levels and an increased risk of T2D. In our study, a hypomethylated CpG site annotated to *TXNIP* showed a faster rate of methylation decline in individuals with T2D compared to NGT individuals, resulting in a larger methylation difference between groups, potentially leading to a higher *TXNIP* gene expression over time. Conversely, *IL18*, an inflammation-induced cytokine that is secreted by immune cells and adipocytes [54], was identified as one of the novel sites in our research, showed a slower decrease in methylation values in individuals with T2D compared to NGT. Inflammation-driven processes in the innate immune system can lead to apoptosis, tissue fibrosis, and organ dysfunction, contributing to insulin resistance, impaired insulin secretion, and renal failure [55]. The changing methylation signatures at these 7 CpG loci over time confirm their responsiveness to variations of diabetes status and suggesting their potential as therapeutic targets for future interventions.

In our follow-up study, we considered the evolving nature of diabetes status and identified seven methylation sites linked to the progression from NGT to prediabetes and T2D: cg23436042, cg11183227 (*MAN2A2*), cg06500161 (*ABCG1*), cg08788930 (*DENND3*), cg11311053 (*NCOR2*), cg06710464 (*BAIAP2*), and cg17058475 (*CPT1A*). *MAN2A2* (2 sites), involved in carbohydrate formation, was linked to fasting insulin in an integrative cross-omics analysis [56]. *DENND3* is a positive regulator of starvation-induced autophagy [57]. *NCOR2* has been identified as a potential target gene for T2D screening in the context of cell-free DNA (cfDNA) methylation changes [58]. It has also been recognized as a potential druggable target for T2D based on an interactome-transcriptome analysis of peripheral blood mononuclear cells (PBMC) in a case-control study of Chinese T2D patients and age- and sex-matched healthy people [59]. *BAIAP2*, the tenth significant site in our study (effect size: 0.94%, *p* value: 2.24 × 10^− 8^), encodes the insulin-responsive protein of 53kDa (*IRSp53*). In our EWAS analysis, we did not identify any CpG sites linked to prediabetes. However, within the progression analysis involving individuals transitioning from NGT to prediabetes or T2D, we observed that 2 out of 7 CpG sites—*MAN2A1* and *ABCG1*—exhibited suggestive significance or nominal significance to prediabetes. This suggests that prediabetes may indeed influence the progression of diabetes from NGT to prediabetes. Our findings reveal that DNA methylation is associated with the progression of diabetes status and the identified CpG sites could serve as valuable biomarkers for tracking disease evolution and guiding personalized treatments. Further investigation with larger sample sizes may be necessary to better understand the epigenetic changes associated with prediabetes.

DNA methylation is a recognized regulator of gene expression. By integrating gene expression data, we identified 104 associations between 40 CpG sites and 96 unique gene transcripts in whole blood. Notably, among the seven CpG sites liked to the diabetes progression, five showed a negative correlation with gene expression levels, including cg23436042, cg11183227 (*MAN2A2*), cg06500161 (*ABCG1*), cg06710464 (*BAIAP2*), and cg17058475 (*CPT1A*), while cg08788930 (*DENND3*) and cg11311053 (*NCOR2*) did not. For instance, methylation at cg06500161 in the *ABCG1* gene was negatively associated with its expression levels, providing evidence for a potential link between hypomethylation at this site and upregulated gene expression, which may contribute to T2D and related diseases. Although methylation at cg19693031, which is annotated to *TXNIP*, was negatively associated with T2D, our analysis in blood did not identify any associations involving the *TXNIP* gene transcript. Prior research has demonstrated that hyperglycemia-induced overexpression of *TXNIP* can lead to pancreatic β-cell apoptosis, cardiomyopathy, and metabolic disorders [46]. However, the EWAS results indicated no significant association between DNA methylation and HOMA-beta function; likely due to the nature of the blood samples used. *TXNIP* gene expression has been found to be upregulated in skeletal muscle samples from individuals with diabetes and prediabetes [55], supporting our hypothesis. As a metabolically active tissue, blood plays a crucial role in the inflammatory and vascular effects associated with adiposity, thus making it relevant to our investigation. Moreover, the advantages of utilizing blood samples include their accessibility, cost-effectiveness, and potential for early diagnosis and treatment, which enhances their practicality for clinical applications.

Our study has notable strengths. Firstly, we have comprehensive CpG site coverage through EPIC and 450k arrays, in contrast to candidate locus studies which typically utilize pyrosequencing methods. Secondly, we conducted a longitudinal analysis spanning seven years, incorporating both DNA methylation profiles and diabetes status assessed, through OGTT in those without a clinical diabetes diagnosis. Lastly, we employed different statistical models to control for potential confounders, thereby enhancing the robustness and reliability of our findings. Our study also has limitations. We did not account for other types of diabetes such as type 1 diabetes and gestational diabetes, which may exhibit different methylation patterns and disease mechanisms. Furthermore, utilizing DNA derived from blood may not completely reflect tissue-specific variations in methylation patterns. Additionally, the lack of a replication cohort from diverse ancestries, focusing solely on individuals of European ancestry, highlights the necessity for future studies to validate our findings across different populations.

## Conclusion

Our study provides new insights into the associations between DNA methylation and T2D through a longitudinal approach involving repeated measurements. We identified novel CpG sites associated with T2D and revealed varying rates of methylation changes at specific loci across different diabetes status groups. Moreover, we underscored the potential of DNA methylation as a biomarker for diabetes progression and demonstrated the relationship between DNA methylation and the gene expression levels.

## Electronic supplementary material

Below is the link to the electronic supplementary material.

**Additional file 1. Text S1:** Selection criteria of individuals in KORA F4 and FF4; **Text S2**: CPACOR Preprocessing Pipeline; **Text S3**: Selection criteria of CpG sites in KORA F4 and FF4; **Text S4**: Quality control for KORA FF4 gene expression data; **Text S5**: WHO criteria of type 2 diabetes. **Fig.S1**: Line plot illustrates the rate of methylation change over time across different groups; **Fig.S2**: Manhattan plots of sensitivity analysis; **Fig.S3**: Venn plot illustrating the overlap of CpG sites from different analysis; **Fig.S4**: The top 10 non-significant pathways associated with T2D and glycemic traits.

**Additional file 2. Table S1:** Significant CpG sites associated with T2D; **Table S2**: Significant differentially methylated regions associated with T2D; **Table S3**: Related diseases or tissues; **Table S4**: Significant CpG sites associated with FPG; **Table S5**: Significant CpG sites associated with HbA1c; **Table S6**: Significant CpG sites associated with HOMA-IR; **Table S7**: Significant CpG sites associated with interaction between T2D and follow-up time; **Table S8**: Significant CpG sites associated with T2D from extended model; **Table S9**: Significant CpG sites associated with T2D from repeated methylation measurements; **Table S10**: Significant CpG sites associated with persistent diabetes; **Table S11**: Significant CpG sites associated with progression of diabetes status from NGT to diabetes; **Table S12**: Associated CpG-transcripts pairs; **Table S13**: Pathways associated to T2D.

## Data Availability

The dataset(s) supporting the conclusions of this article is (are) included within the article (and its additional files). The KORA data are available upon request from KORA Project Application Self-Service Tool (https://www.helmholtz-munich.de/en/epi/cohort/kora); data requests can be submitted online and are subject to approval by the KORA Board.

## Abbreviations

*ABCG1*: ATP-binding cassette sub-family G member 1
*BAIAP2*: BAR/IMD domain containing adaptor protein 2
*B3GNTL1*: UDP-GlcNAc Beta-1,3-N-Acetylglucosaminyltransferase like 1
BMI: Body mass index
*CALR*: Calreticulin
*C20orf3*: Open reading frame 3 encoded at human chromosome 20
*CPT1A*: Carnitine palmitoyl transferase 1 A
CpG: Cytosine-phosphate-guanine
cfDNA: cell-free DNA
*DENND3*: DENN domain-containing protein 3
DMRs: Differentially methylated regions
*EPPK1*: Epiplakin 1
EWAS: Epigenome-wide association studies
FDR: False discovery rate
FPG: Fasting plasma glucose
*FoxK2*: Forkhead Box K2
*FSD1L*: Fibronectin type III and spry domain containing 1 like
GDM: Gestational diabetes mellitus
HbA1c: Hemoglobin A1c
HOMA-B: Homoeostasis model assessment of beta cell function
HOMA-IR: Homoeostasis model assessment of insulin resistance
IFG: Impaired fasting glucose
IGT: Impaired glucose tolerance
*IL18*: Interleukin 18
*ILVBL*: Ilvb acetolactate synthase like
*IRSp53*: Insulin-responsive protein of 53 kDa
KORA: Cooperative Health Research in the Region of Augsburg
*LINE-1*: Long interspersed nucleotide element-1
*MAN2A2*: Mannosidase alpha class 2a member 2
*NCOR2*: Nuclear receptor corepressor 2
NGT: Normal glucose tolerance
OGTT: Oral glucose tolerance test
PCA: Principal component analysis
PCs: Principal components
PBMC: Peripheral blood mononuclear cells
*RARA*: Retinoic acid receptor alpha
SNPs: Single nucleotide polymorphisms
*SREBF1*: Sterol regulatory element-binding transcription factor 1
*SLC1A5*: Solute carrier family 1 member 5
T2D: Type 2 diabetes
*TNXB*: Tenascin XB
*TXNIP*: Thioredoxin-interacting protein
*VARS*: Valyl-tRNA synthetase
WHO: World health organization
*XRCC3*: X-ray repair cross complementing 3
