# Correction: Longitudinal association between DNA methylation and type 2 diabetes: findings from the KORA F4/FF4 study

**DOI:** 10.1186/s12933-025-02760-2

**Published:** 2025-06-14

**Authors:** Liye Lai, Dave Laurence Juntilla, Monica Del C. Gomez-Alonso, Harald Grallert, Barbara Thorand, Aiman Farzeen, Wolfgang Rathmann, Juliane Winkelmann, Holger Prokisch, Christian Gieger, Christian Herder, Annette Peters, Melanie Waldenberger

**Affiliations:** 1 Research Unit Molecular Epidemiology, Helmholtz Zentrum München, German Research Center for Environmental Health (GmbH), Neuherberg, Germany; 2 Institute of Epidemiology, Helmholtz Zentrum München, German Research Center for Environmental Health (GmbH), Neuherberg, Germany; 3 Institute for Medical Information Processing, Biometry, and Epidemiology (IBE), Pettenkofer School of Public Health, Faculty of Medicine, Ludwig Maximilians University, Munich, Germany; 4 German Center for Diabetes Research (DZD), Neuherberg, Germany; 5 Institute of Neurogenomics, Computational Health Center, Helmholtz Zentrum München, Neuherberg, Germany; 6 Institute for Biometrics and Epidemiology, German Diabetes Center, Leibniz Center for Diabetes Research, Heinrich Heine University Düsseldorf, Düsseldorf, Germany; 7 Institute of Human Genetics, School of Medicine, Technical University Munich, Munich, Germany; 8 Cluster for Systems Neurology (SyNergy), Munich, Germany; 9 Chair of Neurogenetics, Technische Universität München, Munich, Germany; 10 Institute for Clinical Diabetology, German Diabetes Center, Leibniz Center for Diabetes Research at Heinrich Heine University Düsseldorf, Düsseldorf, Germany; 11 Department of Endocrinology and Diabetology, Medical Faculty and University Hospital Düsseldorf, Heinrich Heine University Düsseldorf, Düsseldorf, Germany; 12 German Centre for Cardiovascular Research (DZHK), Partner Site Munich Heart Alliance, Munich, Germany

**Correction to:** **Cardiovascular Diabetology (2025) 24:19** 10.1186/s12933-024-02558-8

In the original publication of this article [1], the author noticed the following errors in author names, figure captions and Table 1. The original article has been updated.

The author’s name “Monica Del C Gomez-Alonso” was incorrectly identified as two separate individuals “Monica Del and C Gomez-Alonso”.

Fig. 1 Illustration of the selection criteria for study participants and CpG sites included in the analysis. 

Fig. 2 Miami plot illustrating EWAS results associated with T2D. The x axis indicates the chromosome location, and the y-axis represents the −log10 (*p*-value). The Bonferroni threshold of 1.34×10^−7^ is marked by a red dashed line, while the Benjamini–Hochberg (FDR) threshold (*p_*FDR < 0.05) is indicated by a blue solid line. The upper side represents the positive estimates, and the lower side represents the negative estimates.

Fig. 3 Volcano plots illustrating the results for glycemic traits. The x axis indicates the effect size, and the y-axis represents the −log10 (*p*-value). The Bonferroni threshold of *p*=1.34×10^−7^ is marked by a red dashed line, while the Benjamini–Hochberg (FDR) threshold (*p_*FDR < 0.05) is indicated by a blue dashed line. (A) Volcano plot for FPG. (B) Volcano plot for HbA1c. (C) Volcano plot for HOMA-B. (D) Volcano plot for HOMA-IR.

Fig. 4 Line plots illustrating the rate of methylation change over time for the NGT and T2D groups. The red and blue line represents the individuals with NGT and T2D, respectively. (A) cg19693031 (*TXNIP*); (B) cg00574958 (*CPT1A*); (C) cg15418499 (*IL18*); (D) cg20507228 (*MAN2A2*).

Fig. 5 Venn diagram illustrating the overlap of CpG sites (with annotated gene names) in the sensitivity analysis. The light cyan colour represents the number of significant CpG sites associated with T2D in the main analysis with all individuals. The greyish-yellow colour represents the number of significant CpG sites associated with T2D in the extended models with all individuals. The light pink colour represents the number of significant CpG sites associated with T2D from individuals with repeated methylation measurements at two time points.

Fig. 6 Volcano plots illustrating the association between DNA methylation and changing diabetes status over time. The x axis indicates the effect size, and the y-axis represents the −log10 (*p*-value). The Bonferroni threshold of 2.27×10^−3^ is marked by a red dashed line, while the Benjamini–Hochberg (FDR) threshold (*p_*FDR < 0.05) is indicated by a blue dashed line. (A) Volcano plot for the persistent prediabetes or T2D. (B) Volcano plot for the progression of diabetes.

In Table 1, there are two cohorts, each consisting of four columns. The column titled "T2D N=245" from “KORA F4” cohort has been mistakenly indicated as belonging to “KORA FF4” cohort by a line marker. The correct version of Table 1 has displayed below.

**Table 1:** Characteristics of the study population

Characteristics	KORA F4				KORA FF4			
	All *N* = 1696	NGT *N* = 1113	Prediabetes *N* = 338	T2D *N* = 245	All *N* = 1805	NGT *N* = 1262	Prediabetes *N* = 304	T2D *N* = 239
Age (years)	61 (14)	58 (14)	65 (14)	67 (10)	58 (18)	54.5 (16)	63 (16)	68 (13.5)
Male (%)	828 (48.8%)	499 (44.8%)	184 (54.4%)	145 (59.2%)	868 (48.1%)	554 (43.9%)	172 (56.6%)	142 (59.4%)
BMI (kg/m^2^)	27.5 (5.8)	26.2(5.2)	29.3 (5.7)	30.7(6.7)	27.0 (6.2)	26.0 (5.4)	29.2 (5.2)	30.4 (7.2)
*Smoking*								
Never smoker	710 (41.9%)	460 (41.3%)	156 (46.2%)	94 (38.4%)	746 (41.3%)	522 (41.4%)	118 (38.8%)	106 (44.4%)
Former smoker	737 (43.5%)	462 (41.5%)	156 (46.2%)	119 (48.6%)	766 (42.4%)	517 (41.0%)	138 (45.4%)	111 (46.4%)
Current smoker	247 (14.6%)	189 (17.0%)	26 (7.7%)	32 (13.1%)	293 (16.2%)	223 (17.7%)	48 (15.8%)	22 (9.2%)
Hypertension (%)	772 (45.5%)	377 (33.9%)	198 (58.6%)	197 (80.4%)	646 (35.8%)	317 (25.1%)	159 (52.3%)	170 (71.1%)
Fasting glucose	5.4 (0.9)	5.2 (0.6)	5.8 (0.9)	6.9 (1.9)	5.4 (0.9)	5.2 (0.6)	6.1 (0.8)	7.2 (2.0)
HOMA-IR	2.2 (1.8)	1.9 (1.3)	3.1 (2.5)	5.1 (4.0)	2.1 (1.9)	1.8 (1.4)	3.5 (2.2)	4.8 (4.2)
HOMA-beta	102.0 (65.7)	101.0 (62.7)	110.0 (79.0)	93.5 (97.4)	94.8 (65.5)	93.1 (61.0)	110.0 (87.7)	102. (70.3)
HbA1c	37.0 (6.0)	36.0 (5.0)	38.5 (5.0)	46.0 (12.0)	36.0 (6.0)	34.0 (5.0)	38.0 (5.0)	45.0 (10.8)
HDL-cholesterol	1.4 (0.5)	1.5 (0.5)	1.3 (0.5)	1.2 (0.4)	1.6 (0.7)	1.7 (0.7)	1.5 (0.6)	1.4 (0.5)
Triglycerides	1.3 (0.9)	1.1 (0.8)	1.5 (1.0)	1.7 (1.2)	1.2 (0.8)	1.1 (0.7)	1.5 (1.0)	1.6 (1.2)
Medication	128.0 (7.6%)	0 (0%)	0 (0%)	128 (52.2%)	133 (7.4%)	0 (0%)	0 (0%)	133 (55.6%)
*Parental history*								
Yes	380 (22.4%)	239 (21.5%)	71 (21.0%)	70 (28.6%)	501 (27.8%)	314 (24.9%)	94 (30.9%)	93 (38.9%)
No	773 (45.6%)	582 (52.3%)	135 (39.9%)	56 (22.9%)	1131 (62.7%)	844 (66.9%)	177 (58.2%)	110 (46.0%)
Unknown	254 (15.0%)	159 (14.3%)	53 (15.7%)	42 (17.1%)	173 (9.6%)	104 (8.2%)	33 (10.9%)	36 (15.1%)

Data are median (IQR) for continuous variables and n (%) for categorical variables. The unit for both fasting glucose and HbA1c is mmol/mol. The unit for both HDL-cholesterol and triglycerides is mmol/l. Medication means the glucose-lowering medication
