# Supplementary material for: Longitudinal association between DNA methylation and type 2 diabetes: findings from the KORA F4/FF4 study
Source: Cardiovasc Diabetol. 2025 Jan 18;24:19. doi: 10.1186/s12933-024-02558-8 (PMC11748594; doi:10.1186/s12933-024-02558-8)
Supplement: Supplementary file 1 — Additional file 1. Text S1: Selection criteria of individuals in KORA F4 and FF4; Text S2: CPACOR Preprocessing Pipeline; Text S3: Selection criteria of CpG sites in KORA F4 and FF4; Text S4: Quality control for KORA FF4 gene expression data; Text S5: WHO criteria of type 2 diabetes. Fig.S1: Line plot illustrates the rate of methylation change over time across different groups; Fig.S2: Manhattan plots of sensitivity analysis; Fig.S3: Venn plot illustrating the overlap of CpG sites from different analysis; Fig.S4: The top 10 non-significant pathways associated with T2D and glycemic traits. [file 12933_2024_2558_MOESM1_ESM.docx]

**Text S1 Selection criteria of individuals in KORA F4 and FF4**

The KORA F4 study included 3,080 participants, while the KORA FF4 study involved 2,279 participants. Methylation measurements were available for 1,799 participants in KORA F4 and 1,928 in KORA FF4, using the Illumina 450K Infinium Methylation BeadChip and Infinium MethylationEPIC BeadChip, respectively. Samples with greater than 5% missing values (based on the autosomes only) were removed, as well as whose predicted sex differed from the sex recorded at the time of the interview. After quality control, 1727 individuals remained in KORA F4 and 1874 in KORA FF4.

For this study, individuals with newly diagnosed T2D measured by oral glucose tolerance test (OGTT) or previously known T2D were categorized as having T2D. We further excluded observations due to either other types of diabetes or unknown diabetes status at KORA F4 or FF4. The longitudinal analyses of diabetes status and glycemic and insulin-related traits were restricted to 2,556 participants with at least one DNA methylation measurement at either F4 or FF4. In total, 3,501 observations from 2,556 participants in KORA F4 (1696) and FF4 (1,805) were included in the analysis. Of these participants, 945 (36.97%) had methylation data at both time points.

**Text S2 CPACOR Preprocessing Pipeline**

1. DNA methylation measurement：In the KORA F4 study, genome-wide DNA methylation in whole blood was analysed using the Illumina 450K Infinium Methylation BeadChip (Illumina Inc., San Diego, CA, USA). For the KORA FF4 study, the Infinium MethylationEPIC BeadChip (Illumina Inc., San Diego, CA, USA) was used according to standard protocols provided by Illumina. GenomeStudio software version 2011.1 with Methylation Module version 1.9.0 was used for initial quality control of assay performance and for generation of methylation data export files.
2. Reading in the data: Raw IDAT files were read into R (v4.3.0) using the command read.metharray from the Bioconductor package minfi (v1.46.0) and background corrected using the command bgcorrect.illumina.
3. Sex prediction: When we used the command getSex (minfi v1.46.0) on the raw data. In KORA F4, there was no individuals with a predicted sex different from the sex given at the time of the interview (cut-off -1.5). In KORA FF4, there were two individuals with predicted sex different to the sex given at the time of the interview and these individuals were removed.
4. Quality control on raw intensities: We used the command getQC (minfi v1.46.0) on the raw data. In KORA F4, 1 individual failed the QC (cut-off 9) and was removed. In KORA FF4, individuals were removed whose median intensity was less than 50% of the experiment-wide mean, or less than 2000 arbitrary units (33 individuals).
5. Detection p-value filter: Probes whose detection p-values were greater than 0.01 were set to missing.
6. Sample call rate filter: Samples with greater than 5% missing values (testing the autosomes only) were removed. In KORA F4, this led to the exclusion of 72 individuals. In KORA FF4, 9 individuals were excluded among which 4 individuals were overlapped with those failing raw intensity quality control.
7. CpG call rate filter: In KORA F4, CpG sites with greater than 5% missing values on the autosomes were removed (N= 14541). In KORA FF4, probes with greater than 5% missing values on the autosomes were also removed (N=5786).
8. CpG probe exclusion: In KORA F4, we use the manifest HM450.hg19.manifest.pop.tsv.gz (Population-specific masking HM450 file from https://zwdzwd.github.io/InfiniumAnnotation) and set MASK_general_EUR to TRUE to obtain a reliable list of probes to be excluded. This is based on PMID: 27924034. This yields 59186 CpG sites to exclude. In KORA FF4, 1) Cross-reactive probes: There are publications providing lists for probes that hybridize to multiple possible regions (PMID: 27717381, PMID: 27330998). A total of 44493 unique probes were removed. 2) SNPs within the probe-binding region: The R package minfi v1.28.3 provides a list of SNPs within the probe-binding regions for each CpG. Probes for CpG sites known to be SNPs with minor allele frequency >0.05 (as given by minfi), or probes that had SNPs in the single base extension with minor allele frequency >0.05 were removed (11370 and 5597, respectively).
9. Quantile normalization: Quantile normalization was performed separately on the signal intensities divided into the 6 probe types: type II red, type II green, type I green unmethylated, type I green methylated, type I red unmethylated, type I red methylated (PMID: 25853392). The quantile normalized intensities were then used to generate methylation beta values, a measure from 0 to 1 indicating what percent of the cells were methylated at this locus. This step was performed separately for the autosomes, and for the sex chromosomes. For the sex chromosomes this step was performed separately for men and women. QN was performed using the R package limma v3.56.2 (PMID: 25605792).
10. Blood disorders: In KORA FF4, seven individuals have strong blood disorders. 1 had already been removed due to failing quality control, and the remaining six were removed from the dataset.
11. Cell type heterogeneity: White blood cell type proportions were estimated using the Houseman algorithm (PMID: 22568884) as implemented using the command estimateCellCounts (minfi v1.46.0) on the raw intensities and the default parameters. estimate were performed using the default types: "CD8T", "CD4T", "NK", "Bcell", "Mono",” Gran”.
12. Technical covariates: We calculated the principal components (PCs) of all the non-negative control probes, as per the CPACOR pipeline. Up to 30 control probe PCs can be used as covariates in the regression models to adjust for technical affects. Alternatively, some combination of plate, chip and chip position can be used.
13. Probe count summary: In KORA F4, the original 450K array has 485577 probes, of which 65 are SNP probes for quality control and were removed. Then the array contains 485512 probes (473864 on the autosomes, 11232 on the X chromosome, 416 on the Y chromosome). 59186 were probes to be excluded based on the population-specific masking HM450 file, and 14541 failed the detection p-value filter, a total of 73727. However, some probes overlapped both categories: a total of 70640 were removed. This leaves a total of 414872 probes: 404837 from the autosomes, 9792 from the X chromosome, 243 from the Y chromosome. In KORA FF4, the original EPIC array had 866895 probes, of which 59 are SNP probes for quality control. A “Product Quality Notice” (Tracking Number: PQN0223) issued by Illumina on April 19, 2017, indicated that 977 probes were removed due to underperformance, hence the total of 865859. 40 samples from batch 1 had defective chips and were missing 598 CpG sites. For these individuals the missing CpG sites were simply replaced with missing values in the data. Then the array contains 865859 probes (846232 on the autosomes, 19090 on the X chromosome, 537 on the Y chromosome). 44493 were cross-reactive probes, 11370 and 5597 had SNPs in the CG position and single base extensions respectively, and 5786 failed the detection p-value filter, a total of 67246. However, many probes overlapped multiple categories: a total of 59631 were removed. This leaves a total of 806228 probes: 788106 from the autosomes, 17743 X chromosome, 379 Y chromosome.
14. Sample count summary: In KORA F4, 1799 individuals were measured in one batch using the Illumina HumanMethylation 450 BeadChip. A total of 72 were removed due to quality control: these all failed the detection rate threshold, and 1 additionally failed the median intensity step. This leaves 1727 individuals passing quality control. In KORA FF4, 1928 individuals were measured in two rounds. 2 were removed due to sex mismatch, 33 removed due to failing quality control on the raw intensities and 9 failed the detection p-value filter (4 overlap with intensity filter), leaving 1888 individuals passing quality control. In the first round, there were N=488 KORA FF4 samples. In the second round, there were N=1440 KORA FF4 samples. They were both measured using the Illumina EPIC BeadChip. Seven individuals had a noted strong blood disorder or unusual cell counts, one of whom had already been removed from the dataset. The further 6 individuals were removed. After all these steps, 8 individuals withdrew consent for their data to be used, leaving 1874 individuals.

**Text S3 Selection criteria of CpG sites in KORA F4 and FF4**

Probes with more than 5% missing values on the autosomes were excluded. Additionally, probes containing single nucleotide polymorphisms (SNPs) within the probe-binding regions were removed. Probes were also filtered out if the detection P-value exceeded 0.01, or if they were found to hybridize to multiple genomic regions. Probe intensities were normalized using the quantile normalization procedure for both KORA F4 and FF4. After quality control, 414,872 CpG sites remained in KORA F4 and 806,228 in KORA FF4, with 383,057 overlapping CpG sites. Following the exclusion of sex chromosome CpG sites, 374,054 CpG sites were left in the final analysis.

**Text S4 Quality control for KORA FF4 gene expression data**

After RNA isolation using PAXgene Blood RNA Kit, RNA integrity number (RIN) was measured using the Agilent 2100 Bioanalyzer system. RNA samples with RIN values of approximately 6 or more were selected for mRNA sequencing (poly-A selected). The libraries were prepared using the Illumina stranded mRNA prep ligation kit (Illumina), following the kit's instructions. After a final QC, the libraries were sequenced in a paired-end mode (2x100 bases) in the Novaseq6000 sequencer (Illumina) with a depth of ≥ 40 Million reads per sample. After demultiplexing, FASTQ files from each sample are processed using standard tools. Alignment to UCSC Genome Browser hg19 human reference genome using STAR v2.4.2a (PMID: 23104886). Unaligned reads are discarded. Sequencing QC was done using RNASeQC v1.1.8.1 (PMID: 22539670). Properly aligned reads are then processed with HTSeq-count v0.6.1 (PMID: 25260700) to generate read counts which can be interpreted as quantified gene expression. The reads are then normalized for exon length and total sequencing yield to generate Fragments Per Kilobase of transcript per Million mapped reads (FPKM), and this is done through dividing the fragments per gene by the product of length of the gene in kilobase and million reads sequenced.

After sequencing QC, samples QC was done. Samples with < 30 million reads were discarded. Exonic, intronic, intragenic, intergenic and rRNA rates calculated by RNAseQC were examined for outliers but no such outliers were found, and no samples were excluded based on these. Only the genes with FPKM of ≥ 1 in at least 5% of the samples were selected. Number of the selected genes in each sample were calculated. Samples having less than 5750 genes were excluded. Sex mismatches in the phenotype tables and those discerned from looking at the expression of XIST and UTY genes were also excluded.

**Text S5 WHO criteria of type 2 diabetes**

Normal glucose tolerance (fasting glucose <6.1 mmol/l and 2h glucose <7.8 mmol/l); prediabetes defined as (1) impaired fasting glucose (IFG; fasting glucose ≥6.1 mmol/l but <7.0 mmol/l, and 2h-glucose <7.8 mmol/l), (2) impaired glucose tolerance (IGT; fasting glucose <6.1 mmol/l and 2h glucose ≥7.8 mmol/l but <11.1 mmol/l) or (3) combination of (1) and (2); and newly diagnosed T2D (fasting glucose ≥7.0 mmol/l or 2h-glucose ≥11.1 mmol/l) were defined according to the 1999/2006 WHO criteria.

**Table S1** Characteristics of population with repeated methylation measurements

| **Characteristics** | **KORA F4** | | | | **KORA FF4** | | | |
| --- | --- | --- | --- | --- | --- | --- | --- | --- |
|  | All  N=945 | NGT  N=666 | Prediabetes  N=170 | T2D  N=109 | All  N=945 | NGT  N=570 | Prediabetes  N=194 | T2D  N=181 |
| Age (years) | 57 (12) | 56 (11) | 60 (12) | 64 (12) | 64 (12) | 62 (12) | 65 (11) | 69 (12) |
| Male (%) | 459 (48.6%) | 297 (44.6%) | 97 (57.1%) | 65 (59.6%) | 459 (48.6%) | 239 (41.9%) | 110 (56.7%) | 110 (60.8%) |
| BMI (kg/m2) | 27.1 (5.9) | 26.1 (5.1) | 29.8 (6.0) | 30.4 (6.5) | 27.4 (6.2) | 26.2 (5.6) | 29.0 (5.50) | 29.9 (7.18) |
| Smoking |  |  |  |  |  |  |  |  |
| Never smoker | 389 (41.2%) | 278 (41.7%) | 71 (41.8%) | 40 (36.7%) | 389 (41.2%) | 237 (41.6%) | 77 (39.7%) | 75 (41.4%) |
| Former smoker | 415 (43.9%) | 274 (41.1%) | 84 (49.4%) | 57 (52.3%) | 436 (46.1%) | 256 (44.9%) | 91 (46.9%) | 89 (49.2%) |
| Current smoker | 141 (14.9%) | 114 (17.1%) | 15 (8.8%) | 12 (11.0%) | 120 (12.7%) | 77 (13.5%) | 26 (13.4%) | 17 (9.39%) |
| Hypertension | 367 (38.8%) | 193 (29.0%) | 91 (53.5%) | 83 (76.2%) | 447 (47.3%) | 201 (35.3%) | 114 (58.8%) | 132 (72.9%) |
| Fasting glucose | 5.3 (0.8) | 5.2 (0.6) | 5.9 (1.0) | 7 (2.3) | 5.6 (1.0) | 5.3 (0.6) | 6.1 (0.7) | 7.3 (2) |
| HOMA-IR | 2.04 (1.7) | 1.8 (1.2) | 3.11 (2.5) | 4.61 (3.5) | 2.3 (2) | 2.0 (1.3) | 3.6 (2.2) | 4.8 (4.3) |
| HOMA-B | 99.4 (64.1) | 98.8 (58.4) | 115.0 (78.5) | 84.2 (76.1) | 96.0 (67.7) | 95 (61.9) | 110. (84.2) | 88.4 (71.7) |
| HbA1c | 37.0 (7) | 36 (5) | 38 (4.8) | 46 (11) | 37.0 (6) | 35 (5) | 38 (4) | 45 (10) |
| HDL-cholesterol | 1.4 (0.5) | 1.5 (0.5) | 1.3 (0.4) | 1.2 (0.4) | 1.7 (0.7) | 1.8 (0.7) | 1.5 (0.5) | 1.4 (0.5) |
| Triglycerides | 1.3 (0.9) | 1.1 (0.8) | 1.6 (1.1) | 1.3 (1.2) | 1.3 (0.8) | 1.1 (0.6) | 1.4 (1.0) | 1.6 (1.3) |
| Medication | 46 (4.9%) | 0 (0%) | 0 (0%) | 46 (42.2%) | 104 (11.0%) | 0 (0%) | 0 (0%) | 104 (57.5%) |
| Parental history |  |  |  |  |  |  |  |  |
| Yes | 247 (26.1%) | 161 (24.2%) | 48 (28.2%) | 38 (34.9%) | 268 (28.4%) | 140 (24.6%) | 57 (29.4%) | 71 (39.2%) |
| No | 476 (50.4%) | 365 (54.8%) | 78 (45.9%) | 33 (30.3%) | 569 (60.2%) | 373 (65.4%) | 115 (59.3%) | 81 (44.8%) |
| Unknown | 254 (13.3%) | 90 (13.5%) | 25 (14.7%) | 11 (10.1%) | 108 (11.4%) | 57 (10%) | 22 (11.3%) | 29 (16.0%) |

Data are median (IQR) for continuous variables and n (%) for categorical variables. The unit for both fasting glucose and HbA1c is mmol/mol. The unit for both HDL-cholesterol and triglycerides is mmol/l. Medication means the glucose-lowering medication.


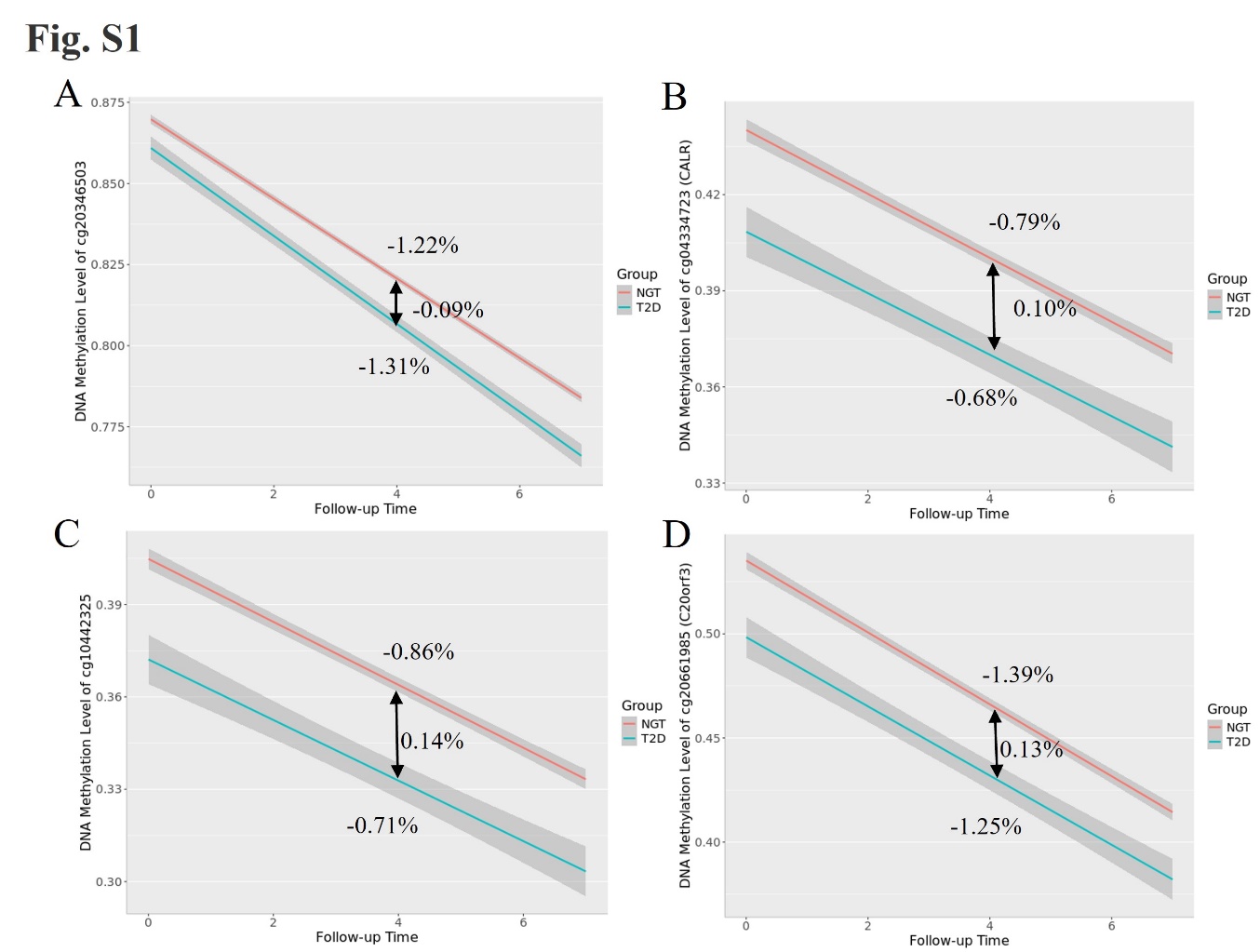


Line plots illustrate the rate of methylation change over time across different groups. The red and blue line represents the individuals with NGT and T2D, respectively. (A) cg20346503; (B) cg04334723 (*CALR*); (C) cg10442325; (D) cg20661985 (*C20orf3*).


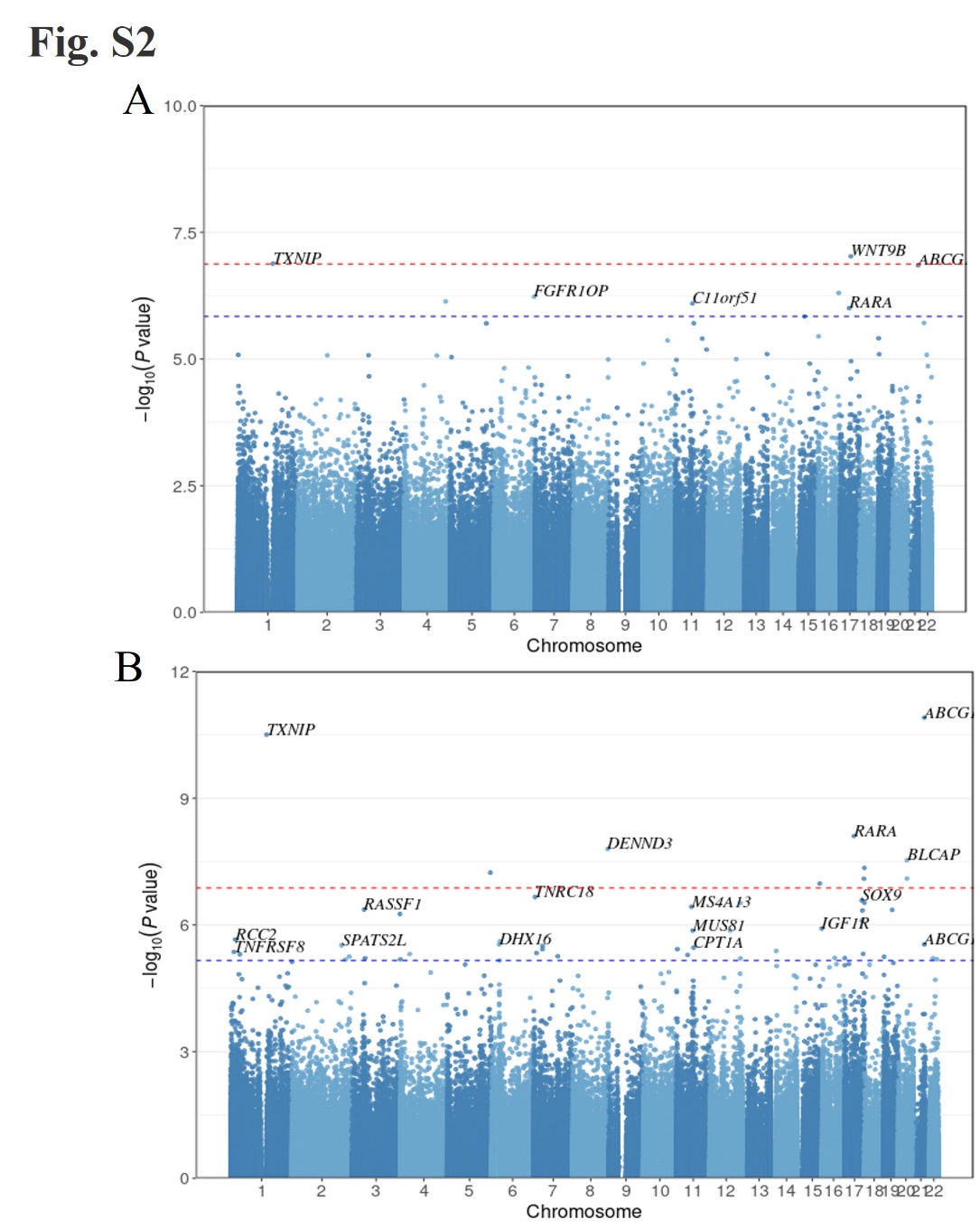


Manhattan plots of sensitivity analysis. The x axis indicates the chromosome location, and the y-axis represents the −log10 (*p*-value). The Bonferroni threshold of 1.34×10^−7^ is marked by a blue solid line, while the Benjamini–Hochberg (FDR) threshold (*p_*FDR < 0.05) is indicated by a red dashed line. (A) Manhattan plot of EWAS results from extended model. (B) Manhattan plots of EWAS results from individuals with two-time points methylation data.


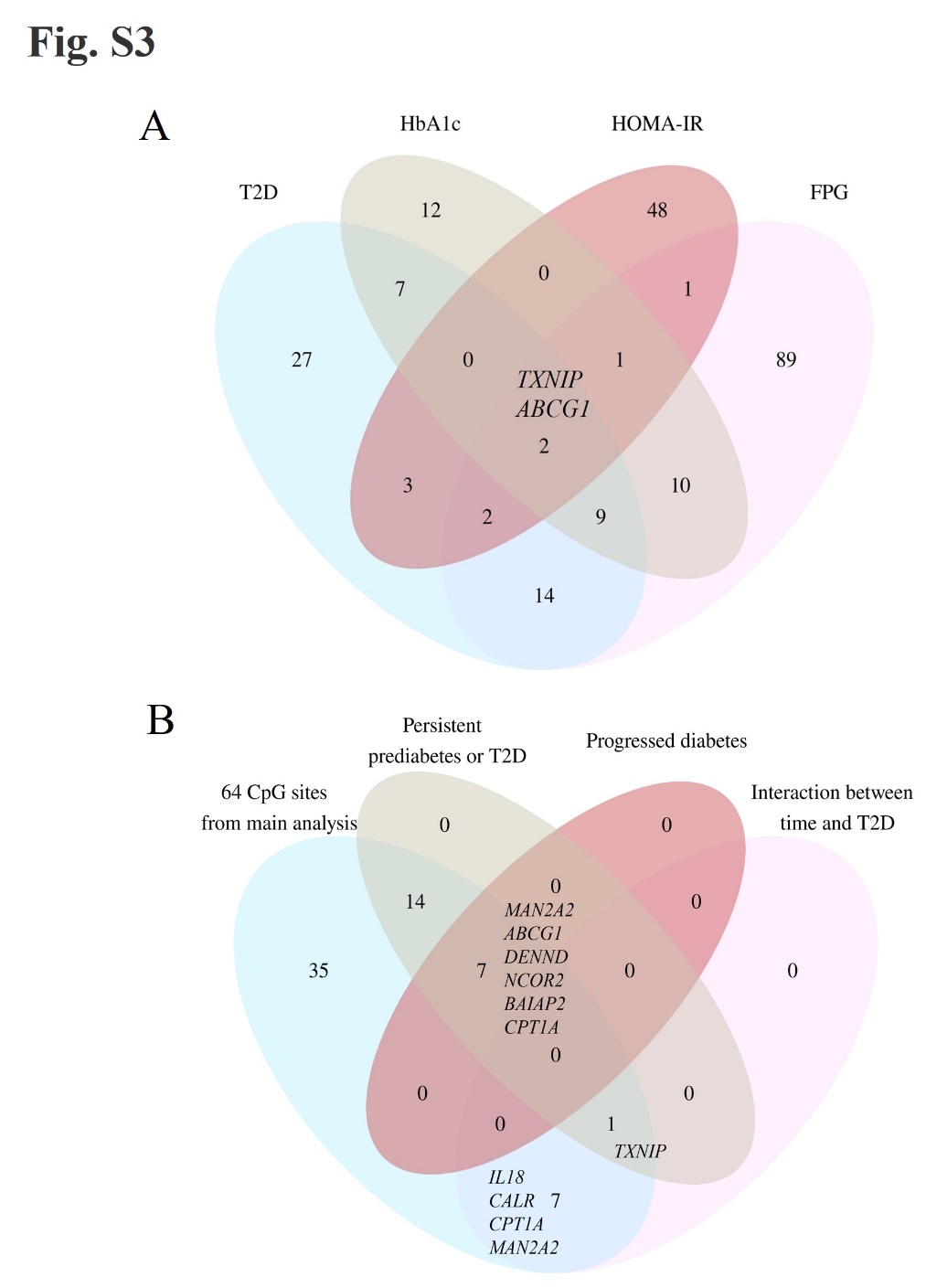


Venn plot illustrates the overlap of CpG sites from different analysis.


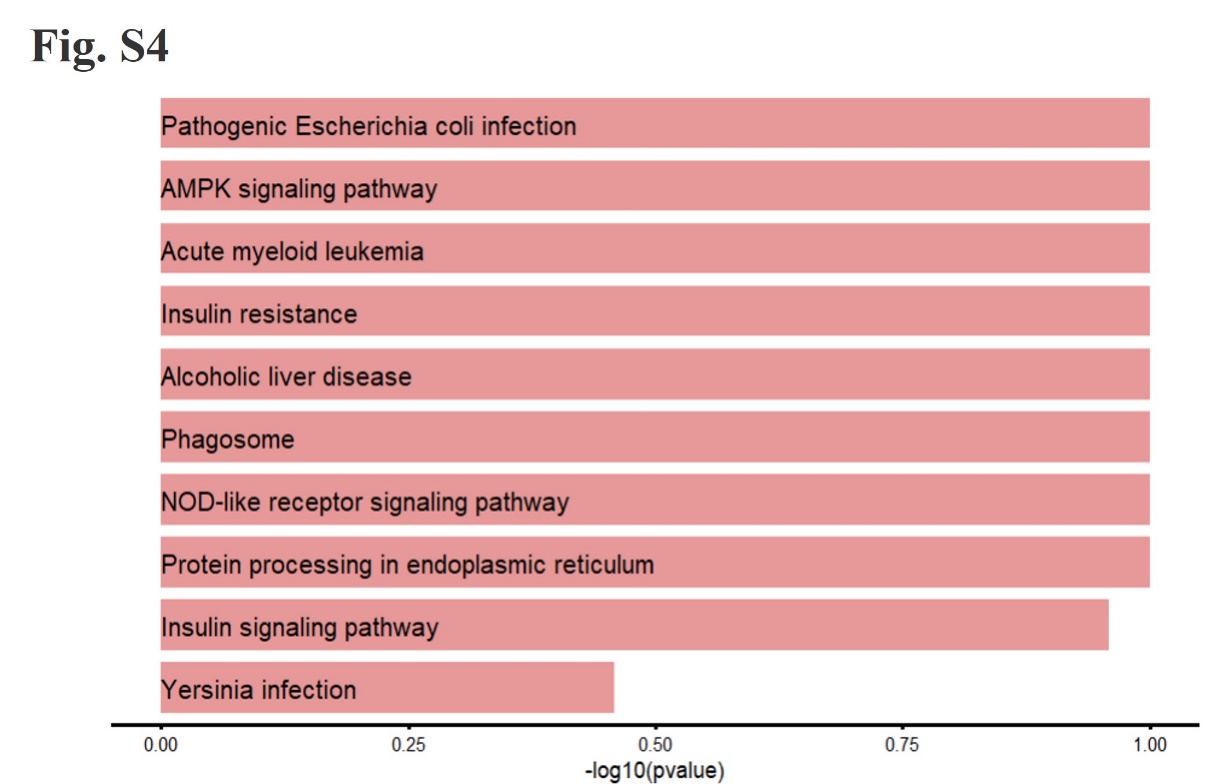


The top 10 non-significant pathways associated with T2D and glycemic traits. The x-axis represents the −log10(p-value), and the red dashed line represents the significant threshold (*p*_FDR < 0.05).
